# DNMBP-AS1/hsa-miR-30a-5p/PGC1α axis suppresses tumor progression of colorectal cancer by inhibiting PKM2-mediated Warburg effect and enhance anti-PD-1 therapy efficacy

**DOI:** 10.1038/s41420-025-02561-2

**Published:** 2025-07-02

**Authors:** Tianxiao Wang, Wenxin Zhang, Jiafeng Liu, Xiang Mao, Xinhai Wang, Jiyifan Li, Yuxin Huang, Zimei Wu, Haifei Chen, Huanying Shi, Huijie Qi, Lu Chen, Qunyi Li

**Affiliations:** 1 Department of Pharmacy, Huashan Hospital, Fudan University, Shanghai, China; 2 Department of Surgery, Huashan Hospital, Fudan University, Shanghai, China

**Keywords:** Colon cancer, Prognostic markers

## Abstract

The Warburg effect, which is aerobic glycolysis, constitutes a major driver of various cancer progression. Therefore, we aimed to examine the role of peroxisome proliferator-activated receptor-gamma coactivator-1α (PGC1α) and its competing endogenous RNA (ceRNA) network in colorectal cancer (CRC) metabolic reprogramming. We used bioinformatics analysis and dual-luciferase reporter gene experiments and identified the DNMBP-AS1/hsa-miR-30a-5p/PGC1α ceRNA network. Additionally, we investigate the impact of PGC1α expression alterations on CRC proliferation and metabolic reprogramming. Moreover, we studied the influence of PGC1α on pyruvate kinase M2 (PKM2), and CRC malignant behavior manifestation. Our study has uncovered a significant association between the DNMBP-AS1/hsa-miR-30a-5p/PGC1α ceRNA network and CRC patient prognosis. Additionally, PGC1α overexpression impeded CRC growth, reduced glycolytic capacity, and enhanced anti-PD-1 therapy efficacy. PGC1α inhibited tumor cell glycolysis by downregulating the WNT/β-catenin pathway depending on peroxisome proliferator-activated receptor gamma (PPARγ), thereby suppressing PKM2. The PPARγ agonist rosiglitazone could hinder CRC proliferation and glycolytic activity. Combined with the PGC1α agonist ZLN005, it exhibits synergistic effects for treating CRC. Moreover, we verified that ZLN005 significantly potentiated PD-1 induced tumor suppression in xenograft mice. Finally, we demonstrated that PGC1α and PKM2 expression patterns in tumor tissues were closely related to patient prognosis. Moreover, we constructed a predictive model to predict the 5-year survival events in CRC patients using random forest model. Our results offer novel perspectives on the role of DNMBP-AS1/hsa-miR-30a-5p/PGC1α network in controlling CRC proliferation, metabolism and immune responses. Furthermore, our investigation reveals that using rosiglitazone combined with PGC1α agonist presents a promising therapeutic approach for managing CRC.

## Introduction

Colorectal cancer (CRC) is a malignancy arising from the epithelial lining cells of the large intestine [1]. It is currently the third most frequently diagnosed malignant tumor worldwide. The 2018 global cancer statistics estimated that CRC has more than 1.8 million new cases and 881,000 deaths, representing 10.2% of total cancer and 9.2% of total deaths [2]. However, CRC shows no apparent symptoms in its early stage; only some patients receive early intervention. Surgical resection is the preferred treatment for patients with resectable colorectal tumors. However, radiation therapy, chemotherapy, and biological and immunological treatments are essential for moderate and advanced CRC patients [3]. The 5-year survival for early-stage CRC patients is ~ 90%, whereas for those with advanced CRC, it is less than 10% [4, 5]. Accordingly, a comprehensive understanding of CRC molecular mechanisms is crucial for developing early diagnosis strategies and efficient therapies.

Metabolic reprogramming is a cancer hallmark accompanied by tumor cell growth [6]. Metabolic reprogramming is a distinctive feature observed in tumor cells, whereby aerobic glycolysis occurs at an elevated rate even in the presence of oxygen, in contrast to the surrounding tissues [7, 8]. The competitive endogenous RNA mechanism (ceRNA mechanism) is based on long-chain non-coding RNA binding to microRNAs (miRs) via competitive sponge attachment, affecting mRNA expression [9]. Exploring lncRNA-miRNA-mRNA ceRNA networks enables the investigation of tumor molecular mechanisms. Different ceRNA networks can regulate the glycolytic pathways of various tumors, including lung cancer, glioma, gallbladder cancer, and osteosarcoma [10–13]. However, identifying pivotal ceRNA networks associated explicitly with CRC development and progression requires further investigation.

Peroxisome proliferator-activated receptor-gamma coactivator-1α (PGC1α) is a transcriptional coactivator that regulates mitochondrial biogenesis and respiration and is expressed in high-energy-demand tissues, including the heart, liver, central nervous system, fatty tissue, and intestinal epithelium [14–16]. PGC1α can govern carcinogenesis, progression, and metabolic status in various cancers, such as renal clear cell, prostate, endometrial, breast cancers, and melanoma, through cooperation with approximately twenty nuclear factors (PPARγ, HNF4, NRF1/2, and ERR) [17–22]. In prostate cancer, PGC1α activates an ERRα-dependent transcriptional program that induces a catabolic state and suppresses metastasis [23]. Conversely, PGC1α promotes breast cancer migration and invasion and increases lung metastasis [21]. CRC studies revealed that PGC1α expression is reduced by ~ 60% in dysplastic mucosal samples compared with normal ones [24]. D’Errico et al. reported that PGC1α mRNA level decreases by 70–90% in dysplastic intestinal mucosa of rats [14]. This suggests that dysfunctional PGC1α contributes to cancer pathologies. However, the regulatory mechanism of PGC1α in CRC progression and aerobic glycolysis remains unclear.

Thiazolidinedione insulin sensitizers (rosiglitazone, pioglitazone, and troglitazone) function by activating PPARγ, enhancing insulin action in target tissues, and improving pancreatic β-cell function. These agents are commonly hypoglycemic agents to manage type 2 diabetes. In 2008 and 2012, Chang et al. and Matteo et al. conducted systematic reviews on the association between rosiglitazone and tumorigenesis, respectively [25, 26]. The results indicated that using rosiglitazone may decrease CRC development risk. Nonetheless, the insufficiency of comprehensive understanding regarding the anti-cancer mechanism of rosiglitazone constrains its application in clinical CRC therapy. Consequently, we investigated the mechanism of rosiglitazone’s anti-CRC properties and searched for combination strategies based on our findings.

This study established a ceRNA (DNMBP-AS1/hsa-miR-30a-5p/PGC1α) network associated with CRC patient prognosis using The Cancer Genome Atlas Program (TCGA) and Gene Expression Omnibus (GEO) datasets. We confirmed the constructed ceRNA network through a luciferase reporter gene experiment. By integrating clinical patient data, we have discovered a significant reduction in PGC1α expression within CRC tissue compared with adjacent non-cancerous tissue. Downregulated PGC1α was associated with poor prognosis. Our findings indicate that PGC1α collaborates with PPARγ to inhibit the Wnt-signaling pathway, suppress glycolytic enzyme PKM2 expression, diminish glycolysis and proliferation, and enhanced the efficacy of immune checkpoint inhibitors in CRC cells. We also demonstrated that the PGC1α-specific agonist ZLN005 combined with the hypoglycemic drug rosiglitazone synergistically treated CRC by inhibiting tumor growth by downregulating the Warburg effect. These findings offer novel insights into the CRC progression molecular mechanisms and suggest activating PGC1α combined with rosiglitazone may be a therapeutic strategy for CRC patients.

## Results

### Microarray analysis identifies the differentially expressed mRNAs and miRNAs that possess prognostic significance with CRC

**Fig. 1:**
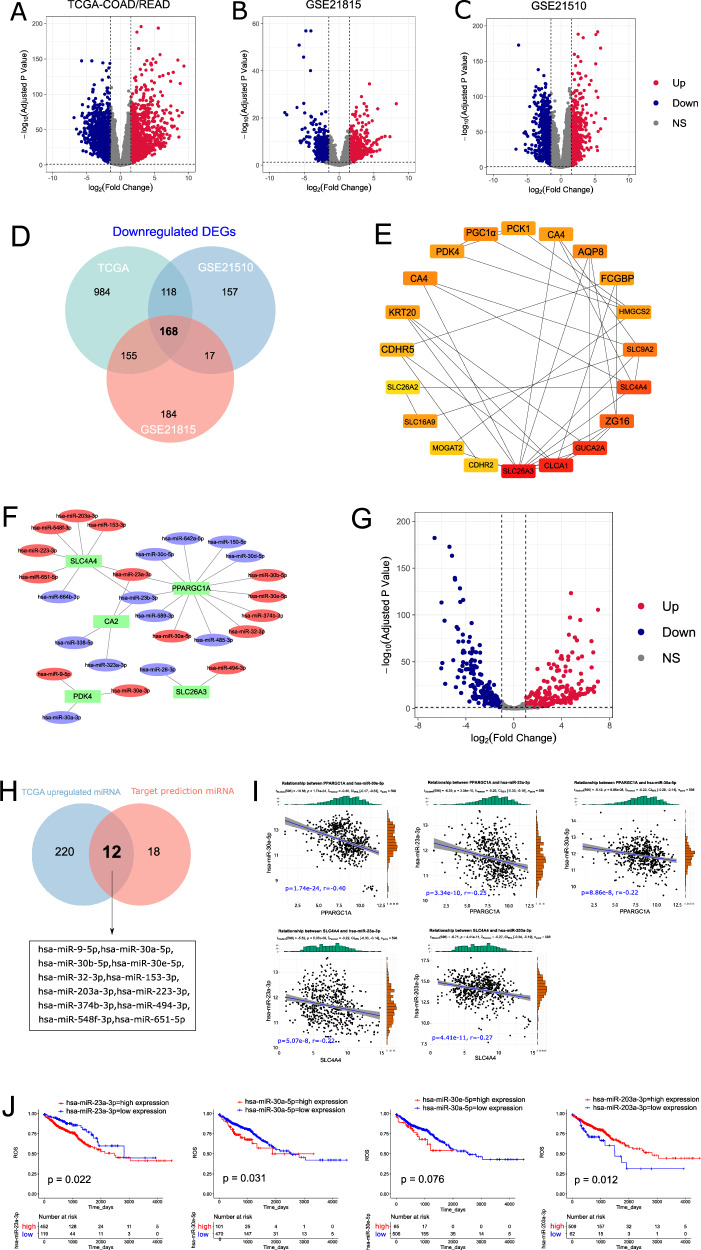
The identification of the differentially expressed mRNAs and miRNAs that possess prognostic significance in colorectal cancer. **A**–**C** Differentially expressed mRNA volcano plot |(log2(fold change)| ≥1.5 or ≤1.5; adj. *p* < 0.05). Blue dots: significantly downregulated (Down); red dots: significantly upregulated (Up); gray dots: no significant differences (NS). **D** The Venn diagram shows the intersection of downregulated genes in three datasets. **E** The PPI network shows the top 20 hub genes of significantly downregulated genes. **F** Construction of a miRNA-mRNA network. The blue and red ellipses represent miRNA. The green diamond indicates downregulated hub mRNA. **G** The volcano plot of differentially expressed miRNAs |log2(fold change)| ≥ 1 or ≤1; adj. *p* < 0.05). **H** Venn diagram shows the intersection between target prediction miRNA (upstream miRNAs of downregulated hub genes) and TCGA upregulated miRNAs. **I** The co-expression analysis of miRNA-mRNA interaction pairs. **J** Overall survival of four miRNA in TCGA-COAD/READ cohort.

We analyzed three mRNA microarray datasets (TCGA-COAD/READ, GSE21815, and GSE21510) to obtain DEGs between CRC and adjacent normal tissues. Based on the volcano plot (Fig. 1A–C), the genes with adj. *P* < 0.05 and |log2(fold change)| >1.5 or < 1.5 were regarded as significant DEGs. The Venn diagram analysis revealed 168 and 138 common downregulated and upregulated DEGs, respectively, in three datasets (Figs. 1D and S1A).

The PPI networks were constructed for the downregulated and upregulated DEGs by the STRING database (http://string-db.org/), respectively, to understand their mutual interactions. The CytoHubba in Cytoscape software results revealed 40 hub genes, 20 of which were downregulated and 20 were upregulated (Figs. 1E and S1B). Furthermore, Kaplan-Meier (KM) plotter analysis using the TCGA database was performed to assess the overall survival (OS) of those hub genes in CRC patients. Four significantly upregulated hub genes (COL1A2, COL11A1, SPP1, THBS2) were identified in CRC and were associated with poor patient prognosis (Fig. S1C–F). Likewise, 13 out of 20 hub genes (AQP8, CA2, CDHR2, CLCA1, FCGBP, GUCA2A, HMGCS2, MOGAT2, PGC1α, SLC4A4, SLC9A2 SLC26A3, and ZG16) were downregulated and were associated with a poor prognosis in CRC. Following the expression pattern and survival analysis, 17 key genes were selected for further analysis.

miRDB database was used to predict the upstream miRNAs of those 17 hub genes, and 26 miRNAs were identified as interacting with five downregulated hub mRNAs based on predicted target score >90. A network of 30 miRNA-mRNA relationships underlying downregulated mRNAs was constructed and visualized by Cytoscape based on mRNA-miRNA regulatory networks (Fig. 1F). However, four downregulated hub mRNAs were ultimately identified as interacting with 23 upstream miRNAs. The mRNA-miRNA regulatory network for the upregulated hub mRNA was constructed and visualized using Cytoscape, which consists of 27 miRNA-mRNA relationships (Fig. S1G).

Next, we examined the differentially expressed miRNAs between CRC tumor and normal samples in the TCGA database, as shown in the volcano plot (Fig. 1G). A total of 231 miRNAs were differentially expressed between CRC and normal tissues based on cut-off criteria (adj. *p* < 0.05 and |log2(fold change)| ≥ 1 or ≤ −1). Based on Venn diagram analyses, 12 of the 30 miRNAs targeted to the hub mRNAs downregulated in CRC were significantly upregulated (Fig. 1H). Furthermore, six miRNAs bound to the upregulated hub mRNAs in CRC were significantly downregulated (Fig. S1H). Using the TCGA-COAD/READ database, the expression correlation was calculated between 18 miRNAs and their target mRNAs by R language (Table S1). Five pairs of miRNAs and mRNAs were inversely correlated (Fig. 1I). According to the OS analysis for patients with CRC, two of the miRNAs (hsa-miR-23a-3p and has-miR-30a-5p) were poor prognostic biomarkers (Fig. 1J).

### Network construction of lncRNA-microRNA-mRNA

**Fig. 2:**
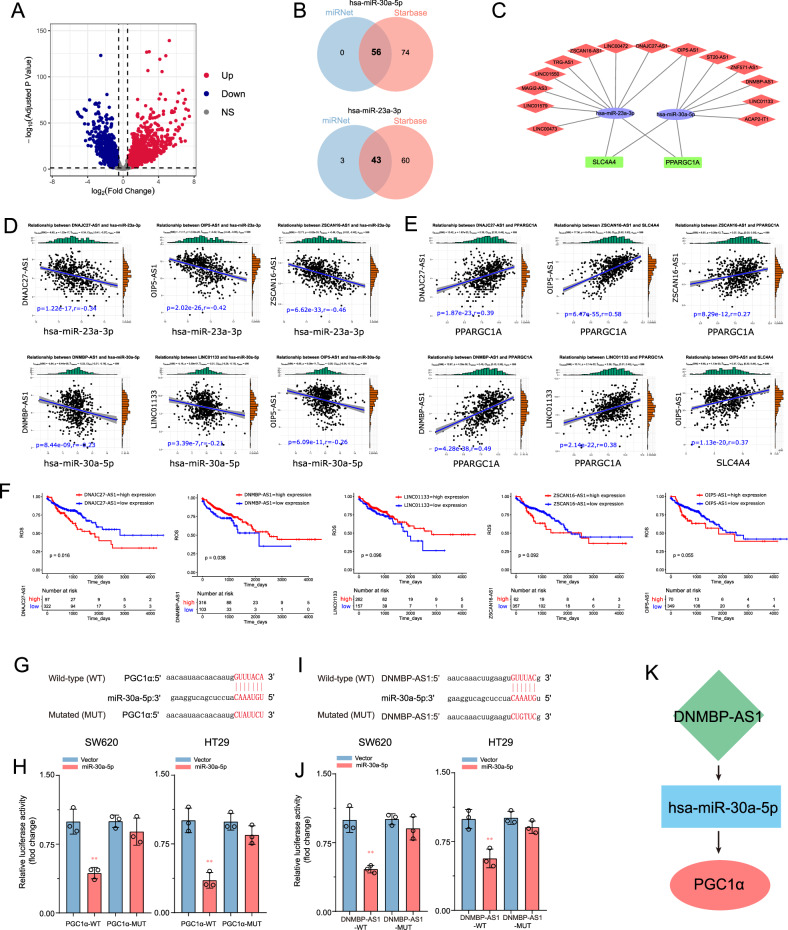
Network construction of lncRNA-microRNA-mRNA. **A** Volcano map of differentially expressed lncRNAs of COAD and READ in TCGA |log2(fold change)| ≥ 0.5 or ≤ 0.5; adj. *p* value < 0.05). Blue dots: significantly downregulated (Down); red dots: significantly upregulated (Up); gray dots: no significant differences (NS). **B** lncRNAs of miRNAs (miR-30a-5p and miR-23a-3p) were identified using miRNet and starBase. **C** The miRNA-lncRNA interaction pairs were visualized using Cytoscape. **D**, **E** Co-expression correlation analysis of miRNA-lncRNA and mRNA-lncRNA. **F** Overall survival of five lncRNA in TCGA-COAD/READ cohort. **G**, **H** The wild-type and the mutated sequences of PGC1α and DNMBP-AS1 mRNA 3’-UTR (mutation site: red). **I**, **J** The luciferase activity of CRC cells (SW620 and HT29) in luciferase reporter plasmid containing wild-type PGC1α 3’-UTR (PGC1α-WT) and mutant PGC1α 3’-UTR (PGC1α-MUT) or wild-type KCNQ1OT1 3’-UTR (KCNQ1OT1-WT) and mutant KCNQ1OT1 3’-UTR (KCNQ1OT1- MUT) co-transfected with miR-30a-5p mimics or negative control was assessed, **p < 0.01. **K** Diagram of the DNMBP-AS1/hsa-miR-30a-5p/PGC1α ceRNA network.

First, we identified lncRNAs that were differentially expressed in CRC patients in the TCGA database and then generated volcano maps using gene expression data with *p* < 0.05 and |log2(fold change)| ≥ 0.5 or ≤ 0.5 (Fig. 2A). In the analysis, 2990 lncRNAs were identified with statistically significant differences, including 1781 upregulated and 1209 downregulated lncRNAs. The upstream potential lncRNAs for hsa-miR-23a-3p and has-miR-30a-5p were then predicted through miRNet2.0 and starBase. Consequently, we generated 15 miRNA-lncRNA interaction pairs (Fig. 2B, C). Using the TCGA database, the expression correlations of 15 pairs of miRNAs and lncRNAs were evaluated in CRC (Table S2), and six significantly negatively correlated miRNA-lncRNA pairs were identified (Pearson’s *R* > –0.2, *p* < 0.05) (Fig. 2D). In parallel, we calculated the expression correlation between 14 lncRNAs and their target mRNAs (Table S3), and five lncRNAs (DNMBP-AS1, DNAJC27-AS1, OIP5-AS1, ZSCAN16-AS1, and LINC01133) demonstrated a significantly positive correlation with their corresponding mRNA (Fig. 2E). OS analysis indicated that high DNMBP-AS1 expression correlates with poor prognosis in CRC patients (Fig. 2F). Eventually, we obtained a ceRNA network (DNMBP-AS1/hsa-miR-30a-5p/PGC1α) associated with CRC prognosis.

Through bioinformatics analysis, we found DNMBP-AS1 may sponges miR-30a-5p to regulate the PGC1α. To further verify the ceRNA hypothesis, luciferase assays were performed to verify the direct interaction between genes. Figure 2G depicts the potential target site for miR-30a-5p. Using dual luciferase reporter assays, we further confirmed that miR-30a-5p directly targets PGC1α. Results demonstrated that miR-30a-5p mimics significantly reduced the luciferase activity of WT PGC1α 3′ UTR (PGC1α-WT) reporter but not the Mut 3′ UTR of PGC1α reporter (Fig. 2H). Transfecting miRNA mimics overexpressed miR-30a-5p in SW620 and HT29 cells, and overexpression efficiency was verified by qRT-PCR (Fig. S2A). Our findings indicated that miR-30a-5p directly targets the 3-UTR of PGC1α to inhibit its expression.

To further investigate whether DNMBP-AS1 functions as a miRNA sponge in CRC cells, we measured miR-30a-5p expression in DNMBP-AS1 knockdown and control SW620 and HT29 cells. In CRC cells, silencing DNMBP-AS1 downregulated PGC1α (Fig. S2B). The miR-30a-5p mimics were transfected into CRC cells containing a luciferase reporter construct containing DNMBP-AS1 (WT or MUT miR-30a-5p binding site) (Fig. 2I). Luciferase assay results show that miR-30a-5p is more likely to enhance the luciferase activity of WT DNMBP-AS1 than MUT DNMBP-AS1 (Fig. 2J). These results indicated that DNMBP-AS1 sponges miR-30a-5p to regulate the PGC1α in CRC cells (Fig. 2K).

### Poor prognosis was associated with low expression levels of PGC1 in CRC tissues

**Fig. 3:**
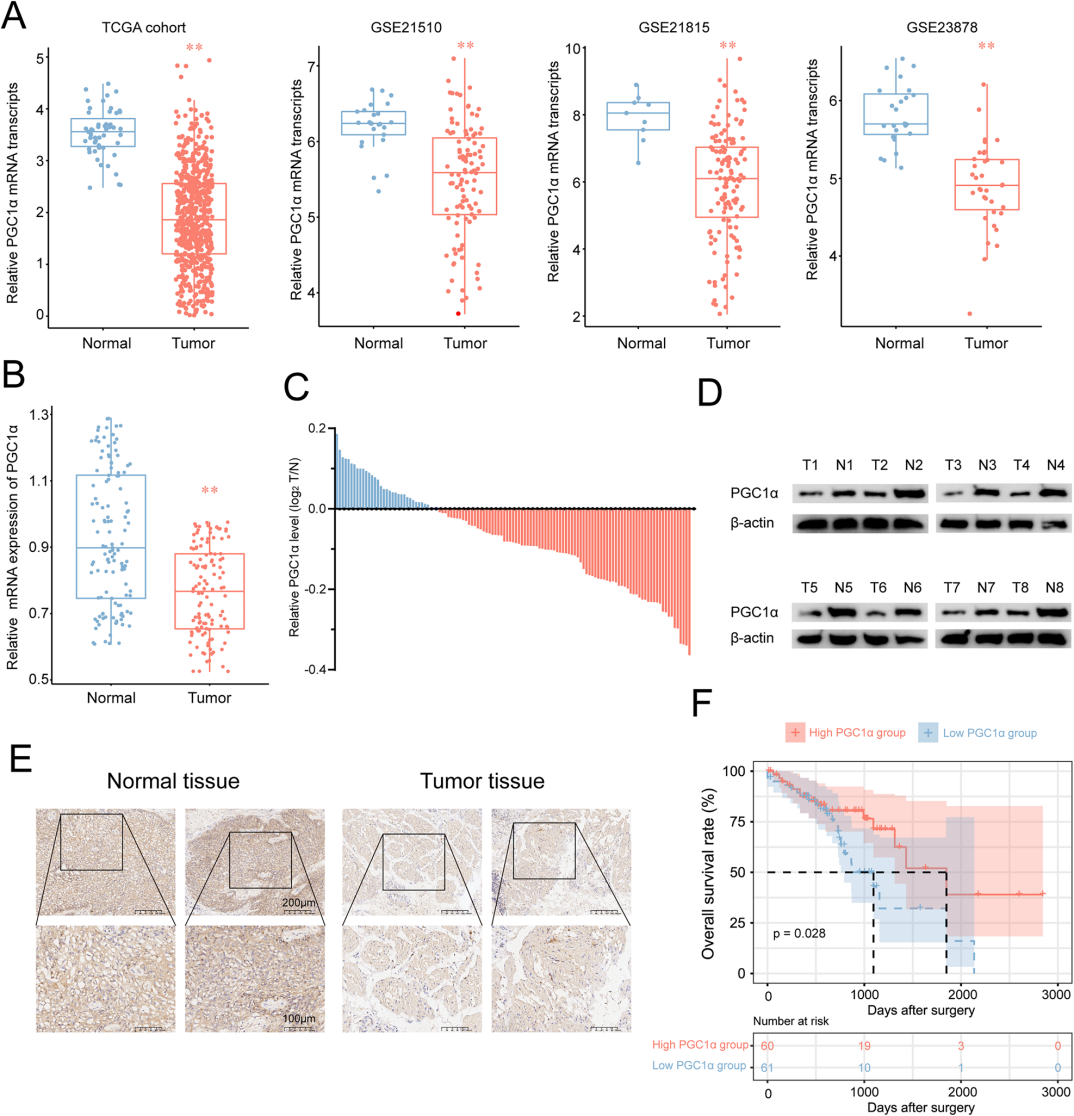
Downregulation of PGC1α and prognostic significance in CRC. **A** PGC1α expression in paired samples of tumor tissues and nontumorous adjacent normal colorectum tissues from patients with CRC in the TCGA and three GSE cohorts. **B**, **C** mRNA levels of PGC1α were evaluated using quantitative real-time PCR in 121 paired samples from patients with CRC. **D** Eight paired samples from patients with CRC were analyzed by Western blot. **E** Immunohistochemical staining for PGC1α was performed on patients with CRC. **F** Kaplan–Meier analysis for OS was performed according to PGC1α mRNA levels. **P < 0.01 indicates significant differences from the normal group as assessed by the Wilcox test.

To investigate the potential involvement of PGC1α in CRC, we analyzed PGC1α expression levels in various datasets, including TCGA-COAD/READ, GSE21510, GSE21815, and GSE23878. Our findings indicated that PGC1α was downregulated in CRC tissues (Fig. 3A). To further elucidate this observation, we performed real-time PCR analysis to assess PGC1α expression levels in human tumors and matched paracancerous tissue samples (121 pairs). Figure 3B, C illustrates that PGC1α was downregulated in 72.7% of the tumor tissues while overexpressed in only 27.3% of the samples compared with the corresponding paracancerous tissue. This result was validated through Western blotting (Fig. 3D) and immunohistochemical staining of tumor sections (Fig. 3E).

To determine the potential clinical relevance of PGC1α downregulation in patients with CRC, we analyzed the mRNA expression of PGC1α in 121 paired samples using qRT-PCR. Next, a KM analysis demonstrated that patients with low PGC1α expression exhibited poor OS compared with those with elevated levels (Fig. 3F). These findings indicate that PGC1α may be a promising clinical biomarker for prognosticating disease outcomes in CRC individuals.

### PGC1α suppressed CRC proliferation in vitro and in vivo

**Fig. 4:**
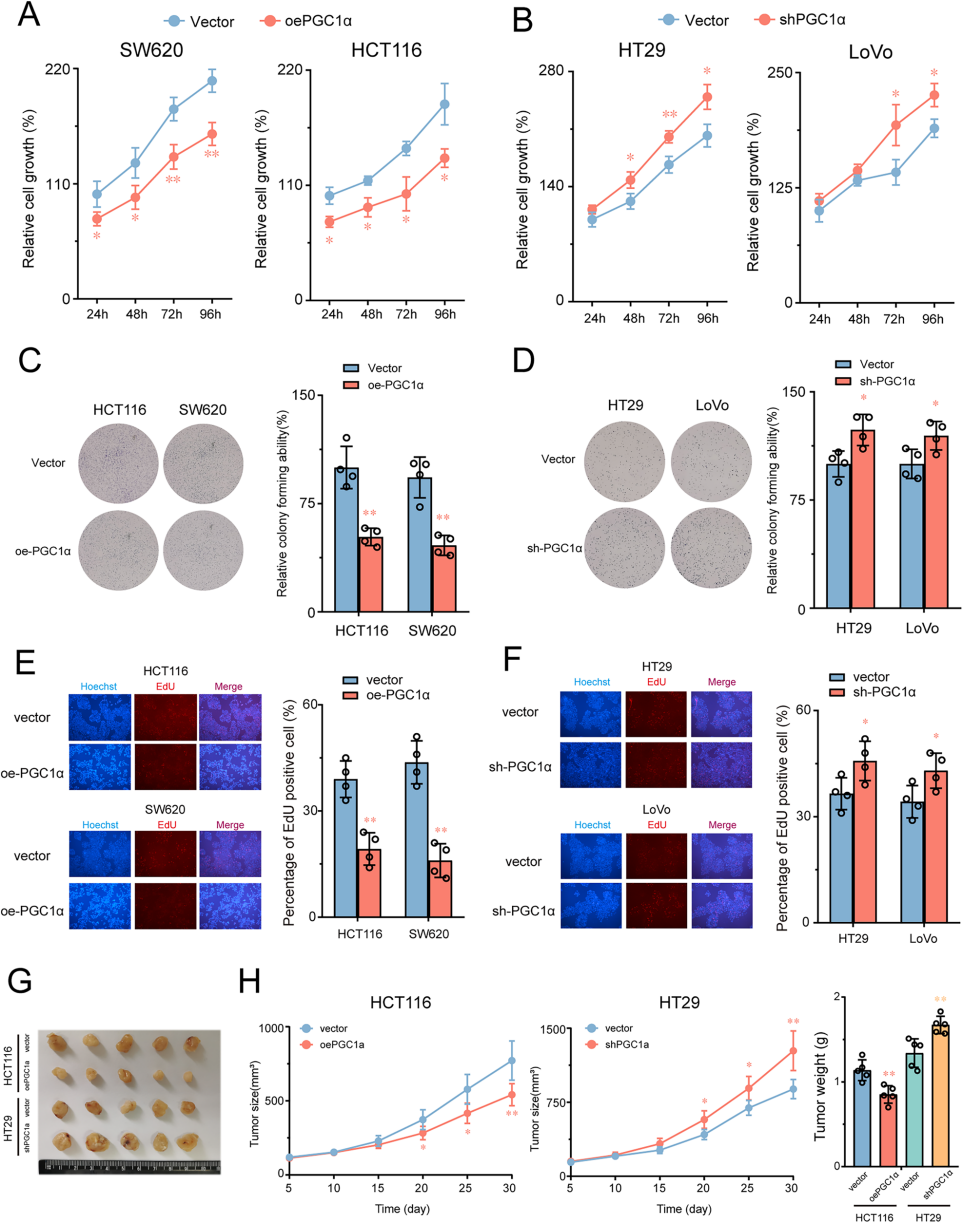
CRC proliferation is suppressed by PGC1α in vitro and in vivo. **A**, **B** Cell viability of CRC cells with PGC1α overexpression or knockdown compared with the control vector group was analyzed using the CCK-8 assay (*n* = 3). **C**, **D** Effects of CRC cells with PGC1α overexpression and knockdown on colony formation compared with control vector group (*n* = 4). **E**, **F** Effects of CRC cells with PGC1α overexpression and knockdown on EDU assay (*n* = 3). **G** Image of tumors isolated from nude mice with tumor xenografts derived from the indicated groups. Effects of PGC1α overexpression or knockdown on tumorigenicity in nude mice. **H** Tumor volumes were monitored at indicated time points. **I** The tumor weight was measured at euthanasia time (*n* = 5). Values are presented as mean ± SD. *P < 0.05 or **P < 0.01 indicates significant differences from the vehicle group as assessed by a one-way ANOVA with a post hoc Dunnett’s test.

To explore the biological function of PGC1α in CRC, we initially analyzed its mRNA and protein expression levels in diverse CRC cell lines (Fig. S3A, B). Subsequently, we generated stable models of PGC1α knockdown in HT29 and LoVo cells and stable models of PGC1α overexpression in HCT116 and SW620 cells based on PGC1α expression levels. The efficacy of these models was evaluated using qRT-PCR and Western blotting (Fig. S4). Significantly, our findings indicate that PGC1α overexpression inhibits CRC cell viability, colony formation, and DNA synthesis (Fig. 4A, C, E) while PGC1α silencing promotes CRC proliferation compared with control cells (Fig. 4B, D, F). To further evaluate the tumor suppressor role of PGC1α in vivo, we subcutaneously transplanted stable cell lines into nude mice and monitored tumor growth. Our results demonstrate that PGC1α overexpression effectively suppresses tumor growth in nude mice compared with the control group, consistent with the in vitro findings. Conversely, suppressing PGC1α significantly increased xenograft tumor growth (Fig. 4G, H). Furthermore, tumor volume and weight were significantly reduced in CRC cells overexpressing PGC1α. In contrast, PGC1α knockdown significantly increased tumor volume and weight compared with the control group. Our in vivo and in vitro results suggest that PGC1α acts as a tumor suppressor in CRC by inhibiting the proliferation of cancer cells.

### PGC1α regulates the Warburg effect to inhibit CRC progression through PKM2 inhibition

**Fig. 5:**
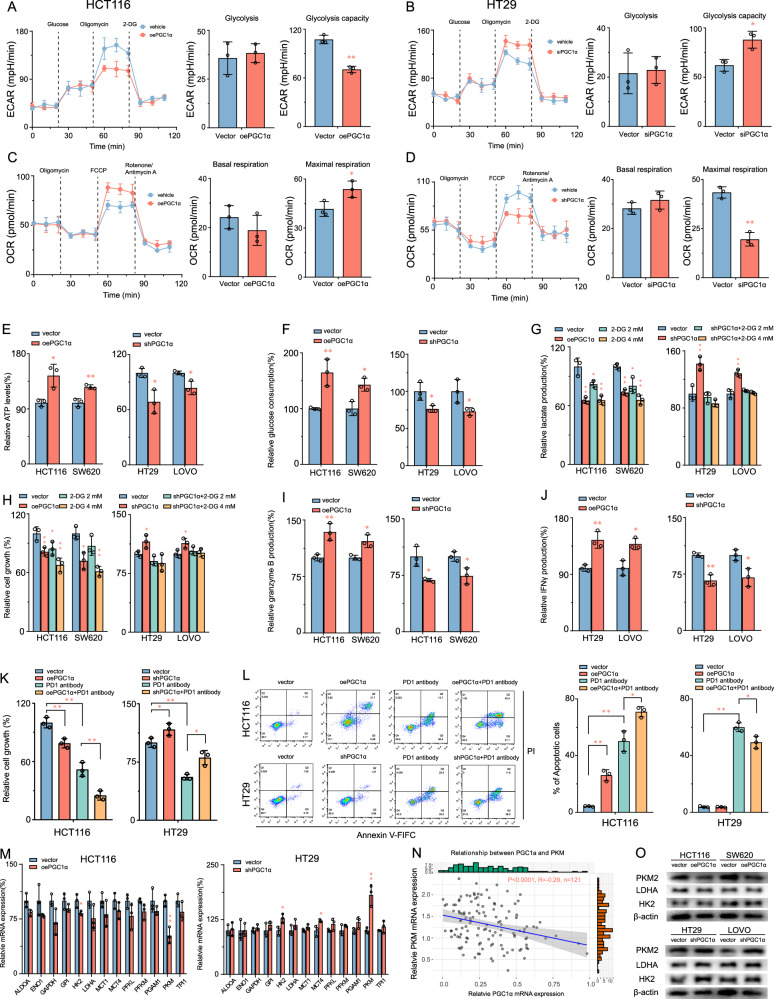
PGC1α regulates the Warburg effect to inhibit CRC progression through PKM2 inhibition. **A**–**D** Effects of PGC1α on oxygen consumption rate (OCR) and extracellular acidification rate (ECAR) in CRC cells. **E**, **F** ATP production and glucose consumption in HCT116 and SW620 cells with PGC1α overexpression and HT29 and LoVo cells with PGC1α knockdown compared with relative control cells. **G**, **H** Effects of 2-DG on the lactate production cell proliferation of CRC cells. **I**, **J** ELSIA assay showed that PGC1α in CRC cells had an effect on the secretion levels of granzyme B and IFN-γ in co-cultured CD8+ T cells. **K**, **L** Effect of PGC1α on the killing ability and immunotherapeutic sensitivity of CD8^+^ T cells in co-culture state. **M** mRNA levels of 13 glycolysis-related genes in HCT116 with PGC1α overexpression and HT29 cells with PGC1α knockdown compared to relative control cells. **N** The correlation between PGC1α and PKM relative levels was determined by RT-PCR in 121 CRC tissues. **O** Effects of PGC1α overexpression and knockdown on PKM2, LDHA and HK2 expression in CRC cells. Values are presented as mean ± SD (*n* = 3). *P < 0.05 or **P < 0.01 indicates significant differences from the vehicle group as assessed by a one-way ANOVA with a post hoc Dunnett’s test.

PGC1α regulates metabolic reprogramming in diverse cancer cells [27, 28]. thereby, it may impede CRC activity. To test this hypothesis, we compared the key metabolic and bioenergetic parameters of PGC1α overexpressing and knockdown CRC cells with those of control cells. We examined the maximal respiration and glycolytic capacity of HCT116 cells overexpressing PGC1α and HT29 cells with PGC1α knockdown. The results indicated that PGC1α overexpression significantly increased the maximal respiration of HCT116 cells by 28.3% while decreasing their glycolytic capacity by 41.3%. Conversely, PGC1α knockdown significantly reduced the maximal respiration of HT29 cells by 15% and elevated their glycolytic capacity by 27% (Fig. 5A–D). Furthermore, our observations indicate a significant increase in intracellular ATP levels and glucose uptake levels in CRC cells that overexpress PGC1α. Conversely, PGC1α depletion declined intracellular ATP levels and glucose uptake while increasing pyruvate production (Fig. 5E, F). These findings suggest that PGC1α regulates cellular energy metabolism in CRC cells.

To further confirm that PGC1α inhibits CRC cell proliferation by regulating the Warburg effect, CRC cells were overexpressed or knocked down for PGC1α and treated with varying concentrations of 2-DG (0, 2, 4 mM) for 48 h, and the lactate production levels and proliferation ability of the CRC cells were evaluated. The results demonstrated that 2-DG significantly and dose-dependently reduced the lactate production levels and the proliferation ability of HCT116 and SW620 cells, similar to the effects of PGC1α overexpression cells. Additionally, 2-DG antagonized the increase in lactate production levels and proliferation ability observed in HT29-shPGC1α and LOVO-shPGC1α cells (Fig. 5G, H). These findings suggest that PGC1α suppresses CRC proliferation by regulating the Warburg effect.

To determine the impact of PGC1α on the functionality of CD8^+^ T cells and the sensitivity of colorectal cancer (CRC) cells to immunotherapy, we conducted co-cultures involving activated CD8^+^ T cells and CRC cells. The outcomes of the enzyme-linked immunosorbent assay (ELISA) revealed diminished production of granzyme B and IFN-γ by CD8^+^ T cells when co-cultured with HCT116 or SW620 cells overexpressing PGC1α. Conversely, CD8^+^ T cells exhibited elevated levels of granzyme B and IFN-γ when co-cultured with HT29 or LOVO cells wherein PGC1α was knocked down (Fig. 5I, J). Following a 72-h co-cultivation of PGC1α-overexpressing HCT116 cells with CD8^+^ T cells, a marked enhancement in the anti-tumor capacity of CD8^+^ T cells and heightened immunotherapy sensitivity of CRC cells were observed. Conversely, co-cultivation of CD8^+^ T cells with PGC1α knockdown HT29 cells resulted in a significant reduction in both the anti-tumor ability of CD8^+^ T cells and the sensitivity of CRC cells to immunotherapy (Fig. 5K, L). This finding substantiates the assertion that PGC1α exerts a discernible influence on the anti-tumor immunity of CD8^+^ T cells.

Next, we utilized qRT-PCR to measure the mRNA levels of 13 vital glycolysis-related genes, revealing that PKM expression levels in HCT116 and HT29 cells changed significantly in response to PGC1α overexpression or knockdown (Fig. 5M). Furthermore, we assessed PKM expression in 121 CRC tissue samples and found a negative correlation between PKM expression levels and PGC1α expression levels (Fig. 5N). Western blot analysis revealed that PKM2 expression was markedly reduced in HCT116 and SW620 cells following PGC1α overexpression, while PGC1α knockdown conversely elevated PKM2 levels in HT29 and LOVO cells. Notably, these manipulations of PGC1α expression exerted no detectable effects on the protein levels of other glycolytic enzymes, including LDHA and HK2 (Fig. 5O). These findings suggested that PGC1α may suppress glycolysis in CRC cells by inhibiting glycolysis-related gene PKM2 expression rather than global glycolysis pathway alterations.

### Inhibition of miR-30a-5p abolished the effect of DNMBP-AS1 on CRC cells

**Fig. 6:**
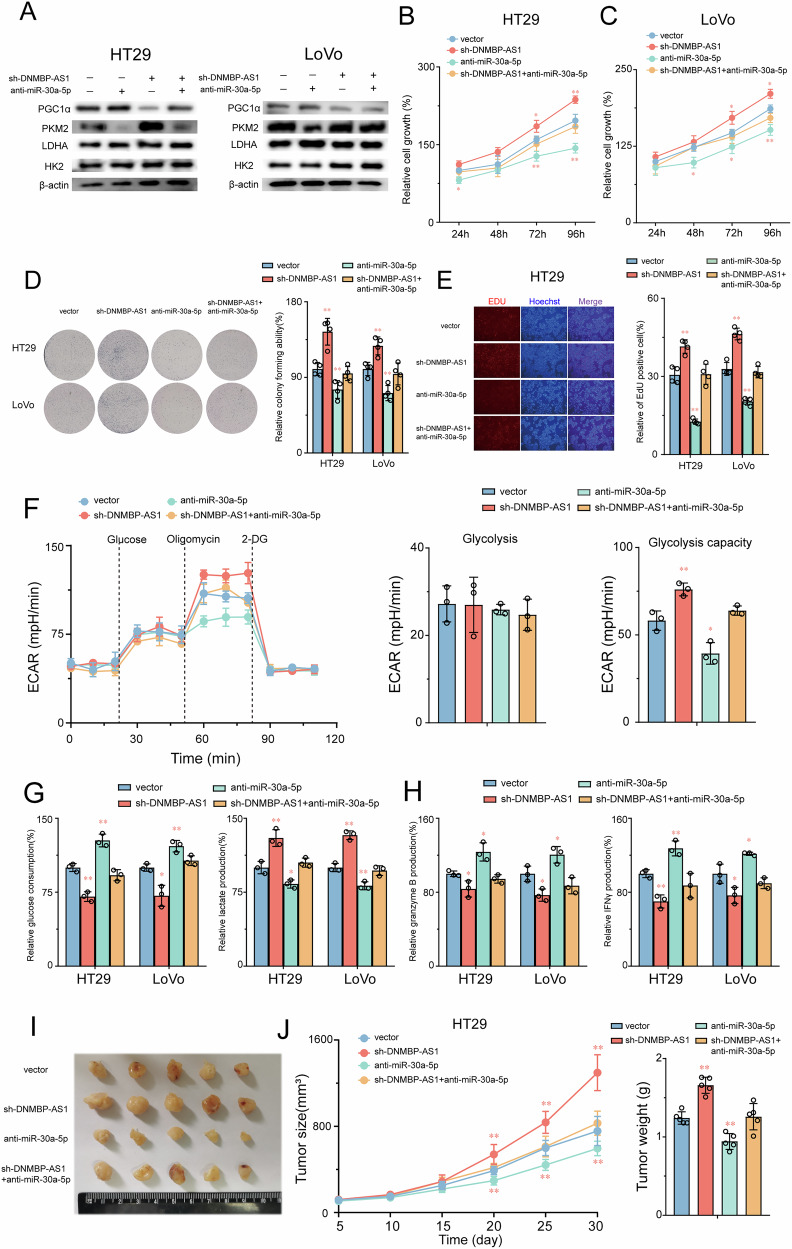
Inhibition of miR-30a-5p abolished the effect of DNMBP-AS1 on CRC cells. **A** Western blot showed the PGC1α, PKM2, LDHA and HK2 expression in HT29 and LoVo cells transfected with anti-miR-30a-5p, sh-DNMBP-AS1, or negative control. **B**–**E** miR-30a-5p inhibitor reversed the promotional effects of DNMBP-AS1-knockdown on the cell viability, colony formation, and DNA synthesis capacity of HT29 and LoVo cells. **F** Altered levels of ECAR and OCR in the HT29 cells in different groups (vector, sh- DNMBP-AS1, anti-miR-30a-5p, and sh-DNMBP-AS1 + anti-miR-30a-5p). **G** Effect of miR-30a-5p/DNMBP-AS1 axis on glucose consumption and lactate production in CRC cells. **H** miR-30a-5p inhibitor reversed the promotional effects of DNMBP-AS1-knockdown on the secretion levels of granzyme B and IFN-γ in co-cultured CD8+ T cells. **I** Typical pictures of tumors isolated from nude mice with tumor xenografts derived from the indicated groups. **J** Tumor volumes were monitored at indicated time points. **J** The weight of tumors was measured at euthanasia time. Values are presented as mean ± SD (*n* = 3–5). *P < 0.05 or **P < 0.01 indicates significant differences from the vehicle group as assessed by a one-way ANOVA with a post hoc Dunnett’s test.

To explore whether the DNMBP-AS1/miR-30a-5p axis regulates CRC cell function through PGC1α, we conducted in vitro and in vivo experiments using sh-DNMBP-AS1 and miR-30a-5p inhibitors in CRC cells. Initially, we found that DNMBP-AS1 downregulation significantly suppressed PGC1α and overexpressed PKM2 in CRC cells, whereas miR-30a-5p inhibition partially reversed the regulatory effect of DNMBP-AS1 knockdown on PGC1α and PKM2 expression; Strikingly, neither LDHA nor HK2 expression exhibited responsiveness to these perturbations of the ceRNA network (Fig. 6A). This selective modulation of PKM2 confirmed the specificity with which the DNMBP-AS1/miR-30a-5p axis governs Warburg reprogramming through targeted PKM2 control rather than global glycolytic pathway modulation. Figure 6B–E illustrates that miR-30a-5p inhibition reversed the promotion of cell viability, colony formation, and DNA synthesis induced by DNMBP-AS1 knockdown. Similarly, miR-30a-5p inhibition partially reversed the increased Warburg effect observed in HT29 cells due to decreased DNMBP-AS1 expression (Fig. 6F, G). The attenuation of DNMBP-AS1 expression markedly curtailed the extrication of GZBM and IFN-γ from CD8^+^ T cells within the milieu of a CD8^+^ T cell co-culture assay. Intriguingly, the imposition of this inhibitory influence was effectively counteracted through the impediment of miR-30a-5p (Fig. 6H). In vivo experiments revealed that DNMBP-AS1 knockdown significantly promoted CRC tumorigenicity, but this effect was abrogated in experimental groups treated with miR-30a-5p (Fig. 6I, J). The findings substantiate the significant regulatory function of DNMBP-AS1/miR-30a-5p/PGC1α axis in the Warburg effect and neoplastic proliferation in CRC cells.

### PPARγ plays a pivotal role in PGC1α-induced inhibition of the WNT/β-catenin pathway and Warburg effect in CRC cells

**Fig. 7:**
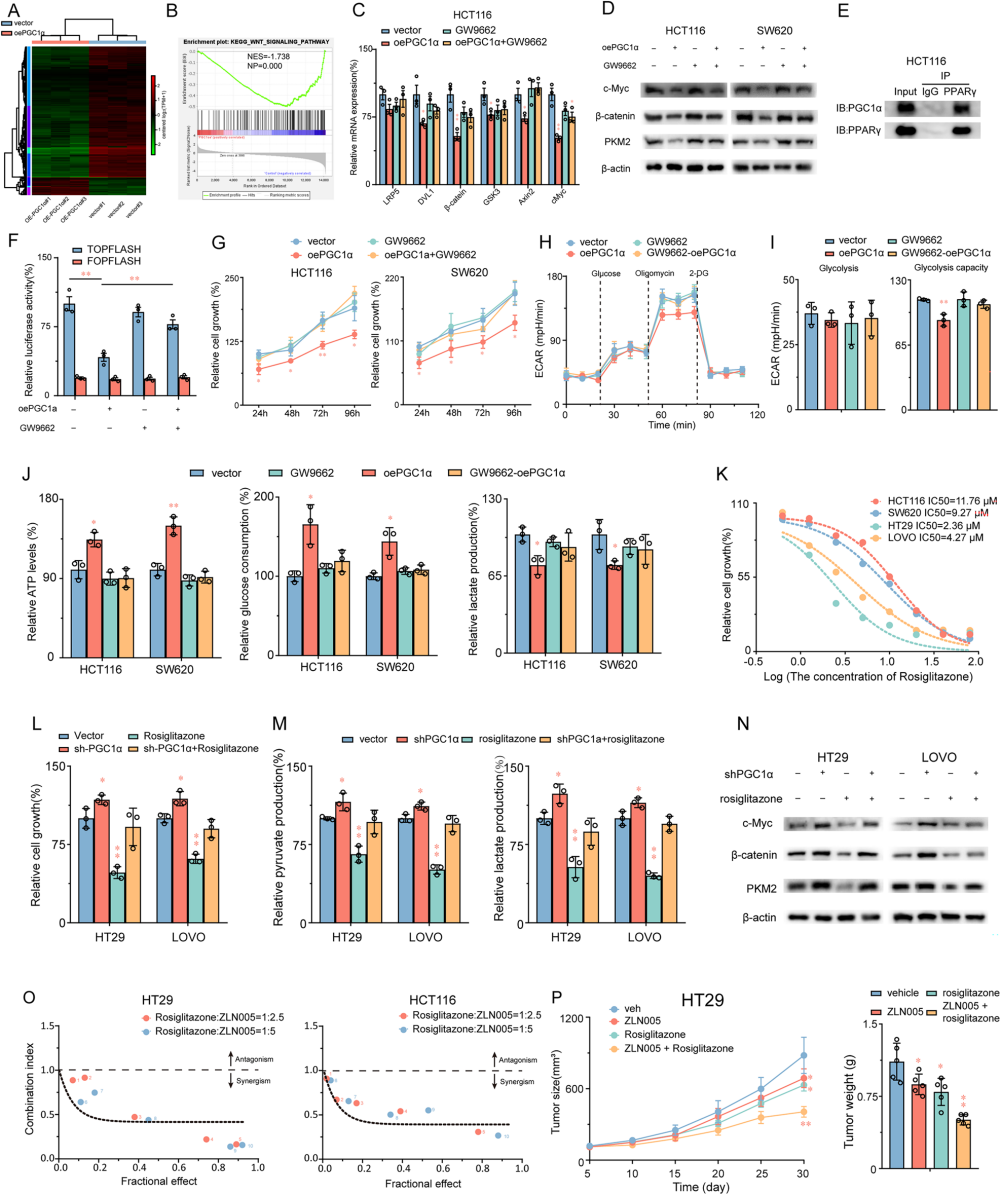
PPARγ plays a pivotal role in PGC1α-induced inhibition of the WNT/β-catenin pathway and Warburg effect in CRC cells. **A** Heatmap illustrates differentially expressed genes in transcript levels between HCT116 cells with PGC1α overexpression and control. **B** Gene set enrichment analysis (GSEA) indicates a significant change in WNT signaling induced by PGC1α. NES, normalized enrichment score. **C** mRNA levels of six Wnt/β-catenin pathway genes in HCT116 with PGC1α overexpression compared with relative control cells. **D** After treatment with GW9662 (10 μM) for 24 h in PGC1α overexpression HCT116 or SW620 cell or WT CRC cells, protein levels of c-Myc, β-catenin, and PKM2 were analyzed using Western blot analysis in the indicated cell lysates. **E** Co-immunoprecipitation assays in HCT116 cells demonstrate direct binding between PGC1α and PPARγ. **F** TOPFlash luciferase reporter assays reveal that PGC1α overexpression significantly reduces β-catenin-driven transcriptional activity in HCT116 cells. **G** GW9662 reversed the inhibition effects of PGC1α overexpression on HCT116 and SW620 cell viability. **H**, **I** Altered levels of ECAR in the HCT116 cells in different groups (vector, oePGC1α, GW9662, and oePGC1α + GW9662). **J** HCT116 and SW620 cells were overexpressed for PGC1α and treatment for GW9662, followed by the determination of ATP production, glucose consumption and lactate production. **K** CRC cells were treated with various concentrations of rosiglitazone for 24 h. The CCK-8 assay determined cell viability. The IC50 value of rosiglitazone in CRC cell lines was then determined. **L** knockdown PGC1α reversed the inhibition effects of PGC1α overexpression on HT29 and LoVo cell viability. **M** HT29 and LoVo cells were knocked down for PGC1α and treatment for rosiglitazone, followed by determination of glucose consumption and lactate production. **N** After treatment with rosiglitazone (10 μM) for 24 h in PGC1α knockdown HT29 or LoVo cell or WT CRC cells, protein levels of c-Myc, β-catenin, and PKM2 were analyzed using Western blot analysis in the indicated cell lysates. **O** HT29 and HCT116 cells were treated for 24 h with indicated concentrations of rosiglitazone plus ZLN005 and then assessed for viability by CCK8 assays. Isobologram analysis shows the synergistic cytotoxic effect of rosiglitazone plus ZLN005. Tumor growth and tumor burdens (**P**) of HT29 xenografts treated with control (vehicle), rosiglitazone or rosiglitazone combined with ZLN005. Values are presented as mean ± SD (*n* = 3–5). *P < 0.05 or **P < 0.01 indicates significant differences from the control group as assessed by a one-way ANOVA with a post hoc Dunnett’s test.

Additional inquiry was performed to examine the mechanisms behind glycolysis inhibition by PGC1α and its regulation of PKM2. The entire genome expression of HCT116 cells was assessed through RNA sequencing. GSEA was performed to ascertain the effect of transcriptional modifications on biological functions and pathways (Fig. 7A, B). Our studies identified a significant inverse regulatory relationship between the WNT/β-catenin signaling axis and PGC1α in colorectal cancer models, as evidenced in HCT116 cells (Fig. 7C). Functional analysis demonstrated that PGC1α overexpression in both HCT116 and HT29 cell lines markedly suppressed key mediators of WNT signaling, including DVL1, β-catenin, GSK3, AXIN2, and c-Myc. Given the established cooperative role of PGC1α and PPARγ in transcriptional regulation, we further probed the mechanistic dependency of this pathway on PPARγ activity. Pharmacological inhibition of PPARγ using GW9662 effectively reversed the PGC1α-mediated suppression of WNT/β-catenin-associated genes and PKM2 expression (Fig. 7D), suggesting a requisite role for PPARγ in this regulatory network. Direct physical interaction between PGC1α and PPARγ was confirmed through co-immunoprecipitation assays in HCT116 cells (Fig. 7E), underscoring their functional partnership as transcriptional co-regulators. To delineate the downstream consequences of this interaction, we employed a Wnt/β-catenin-responsive luciferase reporter system (TOPFlash), revealing that PGC1α overexpression substantially attenuated β-catenin-driven transcriptional activity-a phenomenon entirely abolished by pretreatment with the PPARγ antagonist GW9662 (Fig. 7F). These integrated findings establish that PGC1α exerts its tumor-suppressive effects in colorectal cancer through PPARγ-dependent modulation of WNT/β-catenin signaling. Furthermore, the inhibitory effect of PGC1α on CRC proliferation was effectively blocked by GW9662 (Fig. 7G). GW9662 also reversed the reduction in glycolytic capacity, glucose consumption, and lactate production levels induced by PGC1α overexpression in CRC cells (Fig. 7H, J). These findings prove that PPARγ and PGC1α collaboratively inhibit WNT/β-catenin signaling, suppress glycolysis, and inhibit proliferation in CRC cells.

Prior studies have established the crucial regulatory function of PPARγ in the anti-CRC properties of PGC1α. Accordingly, our study aimed to assess the inhibitory impact of PPARγ selective agonist, rosiglitazone, on CRC proliferation. Herein, we examined the effects of various concentrations of rosiglitazone (0, 0.625, 1.25, 2.5, 5, 10, 20, 40, and 80 μM) on CRC cell viability over 24 h. Our results revealed that the IC50 values for HCT116, SW620, HT29, and LOVO cells were 11.76, 9.27, 2.36, and 4.27 μM, respectively (Fig. 7K). Furthermore, PGC1α knockdown significantly counteracted the inhibitory effects of rosiglitazone on CRC cell proliferation, pyruvate, and lactate production (Fig. 7L, M). Rosiglitazone significantly inhibited c-Myc, β-catenin, and PKM2 protein expression in CRC cells, and knocking down PGC1α reversed this effect (Fig. 7N). These findings suggest a close relationship between PGC1α expression and the inhibitory effects of rosiglitazone on CRC proliferation.

Therefore, we sought to determine whether the combined rosiglitazone and the PGC1α agonist ZLN005 would synergistically treat CRC. We applied Chou-Talalay isobologram analysis to evaluate this combined effect, revealing that administrating rosiglitazone and ZLN005 to CRC cells in a gradient concentration for 24 h resulted in a combination index (CI) of less than 1. This indicates a synergistic effect between the two compounds (Fig. 7O). The findings demonstrate that the co-administration of rosiglitazone and ZLN005 resulted in a synergistic anti-CRC activity with a CI value of less than 1.

In the nude mice xenograft experiment, the combined ZLN005 and rosiglitazone significantly inhibited tumor volume and weight in mice with tumors more than monotherapy (Fig. 7P). Recently, numerous glycolysis inhibitors have demonstrated anti-tumor activity. The co-administration of rosiglitazone and PGC1α agonist significantly inhibited CRC proliferation, indicating that inhibiting glycolysis through the combined rosiglitazone and PGC1α agonist effectively inhibited CRC growth. These results demonstrate that combining rosiglitazone and PGC1α agonists may be a new therapeutic strategy for treating CRC.

### ZLN005 augments the CD8^+^ T cell-mediated antitumor immune response in CRC

**Fig. 8:**
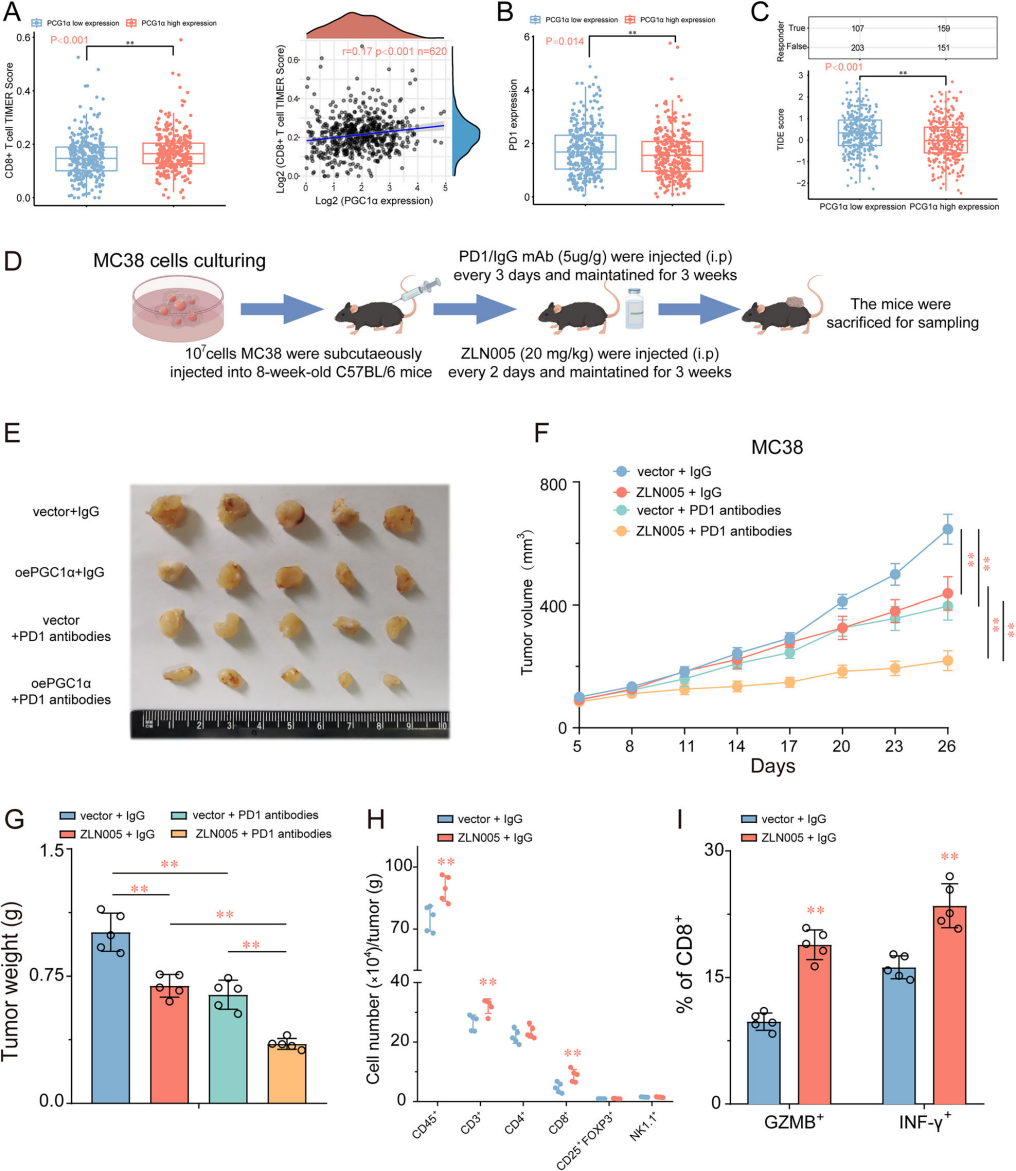
ZLN005 augments the CD8^+^ T cell-mediated antitumor immune response in CRC. **A** TIMER algorithm employed to examine the correlations between PGC1α expression and CD8 + T cells immune infiltration score. **B** PD1 expression levels were lower in the PGC1α high expression group. **C** TIDE scores were lower in the PGC1α high-expression group. **D** Schematic diagram of administration cycles in mice. Tumor growth (**E**, **F**) and tumor burdens (**G**) of MC38 xenografts treated with control (vehicle) or ZLN005 combined with IgG2a or anti–PD-1 mAb (*n* = 5). **H**, **I** Quantification of tumor-infiltrating immune cells, GZMB^+^ CD8^+^ cells and IFN-γ^+^ CD8^+^ cells analyzed by flow cytometry using the indicated cell surface markers and in control and ZLN005 group. *P < 0.05 or **P < 0.01 indicates significant differences from the vehicle group as assessed by a one-way ANOVA with a post hoc Dunnett’s test.

In the preceding section, our findings evinced that the augmentation of PGC1α through overexpression enhances the antitumor immune responses of CD8^+^ T cells within a co-culture system. We hypothesized that specific agonists targeting PGC1α might similarly possess the capability to amplify the anti-tumor immune effects in conjunction with immune checkpoint inhibitors. We scrutinized the association between the expression of PGC1α within the TCGA-CODA/READ dataset and immune scores utilizing TIMER2.0 (http://timer.cistrome.org/). As delineated in (Fig. 8A), the CD8^+^ T cell TIMER score exhibited a significant elevation in the PGC1α high expression cohort compared to their PGC1α low expression counterparts. Noteworthy was the discernible positive correlation between the PGC1α expression level and the CD8^+^ T cell TIMER score. Furthermore, an observation emerged in Fig. 8B, showcasing a lower PD1 expression level in the PGC1α high expression group in comparison to the PGC1α low expression group. This observation implies a relatively weaker immunosuppressive effect in the presence of heightened PGC1α expression. Considering that Tumor Immune Dysfunction and Exclusion (TIDE) scores serve as indicators of sensitivity to immune checkpoint inhibitors, an evaluation of disparities in TIDE scores between the PGC1α high expression and low expression groups within the TCGA-COAD/READ dataset ensued. Our outcomes conclusively manifested a significantly diminished TIDE score in the PGC1α high expression cohort relative to the low expression group (Fig. 8C). This suggests that patients exhibiting heightened PGC1α expression may manifest superior efficacy when subjected to immune checkpoint inhibitors.

Building upon these findings, we delved into an additional investigation to indicate whether targeting PGC1α could enhance the antitumor efficacy of PD-1 blockade. Our outcomes substantiated that sole ZLN005 treatment at a dose of 20 mg/kg significantly attenuated tumor growth and weight in mice bearing MC38 tumors. As anticipated, we also noted that monotherapy with anti-PD-1 (100 µg) had an impact on both tumor volume and weight (Fig. 8D). Significantly, the combination of ZLN005 targeting and anti-PD-1 therapy resulted in additional enhancements in therapeutic benefits compared to either monotherapy strategy (Fig. 8E–G). Subsequently, we gathered MC38 tumor samples for supplementary analysis. Flow cytometry analysis revealed that ZLN005 treatment significantly increased CD8 + T cell infiltration in MC38 tumors (Fig. 8H). This effect correlated with reduced PD-1 expression in the PGC1α-high group, suggesting a potential interplay between metabolic reprogramming and immune checkpoint modulation. Additionally, ZLN005 enhanced the secretion of granzyme B (GZMB) and IFN-γ by CD8^+^ T cells (Fig. 8I), indicative of improved effector function. Cumulatively, our findings furnish compelling evidence that targeting PGC1α may constitute a promising therapeutic strategy to enhance the efficacy of anti-PD-1 therapy in CRC.

### Predicting the 5-year survival probability of colorectal cancer patients using PGC1α-PKM expression patterns combined with machine learning algorithms

**Fig. 9:**
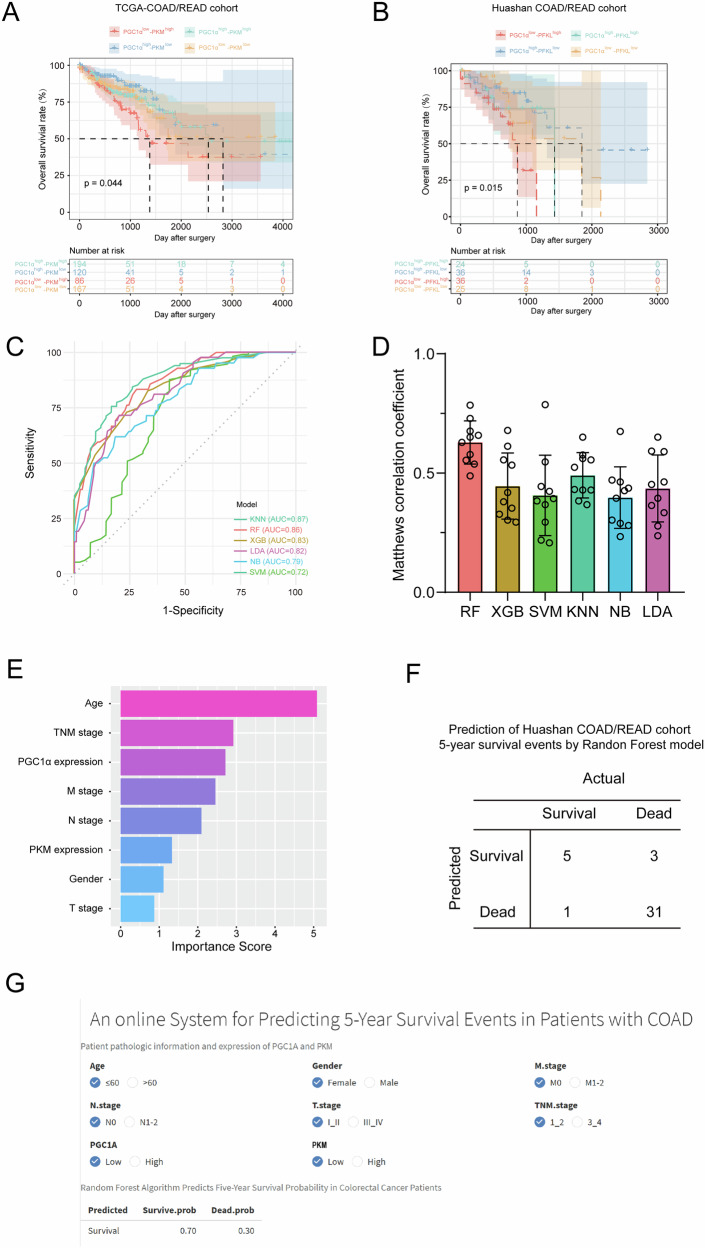
Predicting the 5-year survival probability of colorectal cancer patients using PGC1α-PKM expression patterns combined with machine learning algorithms. **A**, **B** The prognostic value of combining PGC1α and PKM levels was analyzed by Kaplan–Meier analysis in the TCGA-COAD/READ cohort and Huashan COAD/READ cohort. **C**, **D** ROC curve analysis and Matthews correlation coefficient of six machine learning algorithms for prediction the 5-year survival probability of COAD patients in the TCGA-COAD/READ cohort. **F** Prediction of the 5-year survival events for Huashan COAD/READ cohort patients using the established random forest model. **E** The web-based calculator for predicting 5-year survival events in patients with colorectal cancer.

To explore the prognostic values of the PGC1α-PKM axis in CRC patients, the TCGA-COAD/READ cohort and Huashan COAD/READ cohort were analyzed for PGC1α and PKM expression levels to determine their prognostic value. KM analysis demonstrated that CRC patients with high PGC1α expression and low PKM expression had the highest OS (Fig. 9A, B). To better understand the prognostic significance of PGC1 and PKM expression patterns in CRC patients, we employed six machine learning algorithms models to predict the occurrence of 5-year survival events in CRC patients. We screened patients within the TCGA-COAD cohort, identifying individuals who survived for more than five years and those who succumbed within this timeframe (a total of 156 patients). These groups were categorized as 5-year survival success (Alive, 42 patients) and 5-year survival failure (Dead, 114 patients). Subsequently, the pertinent clinical data and PGC1α and PKM expression in these individuals were extracted and systematically arranged to construct six machine learning algorithms models to predict 5-year survival event occurrence in CRC patients, employing the mlr3 package of R programming language.

Comparisons of the performance of prediction among the six models in TCGA-COAD cohort are detailed in (Fig. 9C, D). It turned out that the random forest model demonstrated the highest performance of predicting 5-year survival event, whose AUC was 0.860, matthews correlation coefficient was 0.63. Accordingly, we chose the random forest model as the final prediction model.

According to the feature importance derived from the random forest model, the significance of each clinical parameters was determined by aggregating the feature importance values obtained from each parameter. Figure 9E presents the ranking of clinical parameters’ importance based on the random forest model. The findings indicate that, within the random forest model, the PGC1α and PKM gene expressions were ranked third and sixth in terms of their importance scores, respectively. To ascertain the model efficacy, we screened patients from the Huashan-COAD cohort who survived for more than five years and those who succumbed within five years (40 cases). Subsequently, we employed the pre-existing decision tree model to predict the 5-year survival outcomes for these 40 patients. By comparing these predictions with the clinical follow-up data, we observed that our constructed model achieved a validation cohort prediction accuracy of 90% (Fig. 9F). Furthermore, we have developed an internet-based computational tool utilizing the most effective models (https://huashanpharmacy.shinyapps.io/COAD_PGC1A), enabling healthcare professionals to estimate the likelihood of a patient’s five-year survival by conveniently entering the patient’s pathologic variables and the expression levels of PGC1α and PKM (Fig. 9G).

## Discussion

Cancer cells use glycolysis or oxidative phosphorylation (OXPHOS) to adapt to changing nutrition and oxygen supplies due to many pressures and environmental conditions. PGC1α, an important regulator of cellular metabolism, plays a crucial role in cancer by modulating mitochondrial biogenesis and oxidative metabolism [29]. Moreover, PGC1α is involved in tumorigenesis, growth, metastasis, and apoptosis [17] and exerts a cancer-promoting effect in breast cancer and a cancer-suppressing effect in prostate cancer [21, 23]. Although the regulatory impact of PGC1α on tumorigenesis and development may differ depending on the tumor type, its role in CRC remains unclarified. Herein, we have successfully established and validated a ceRNA network (DNMBP-AS1/hsa-miR-30a-5p/PGC1α) that regulates CRC development. Our findings have revealed the underlying mechanism by which PGC1α inhibits CRC proliferation and aerobic glycolysis. Furthermore, we have investigated the synergistic effect of PGC1α agonists and PPARγ agonist rosiglitazone in treating CRC. Our results have contributed to a better understanding of the molecular mechanisms of CRC progression and elucidated the potential role of PGC1α as a tumor suppressor in CRC development and progression.

Our study is the first to describe the regulatory ceRNA network involving DNMBP-AS1/hsa-miR-30a-5p/PGC1α and its role in regulating CRC growth and aerobic glycolysis. The hsa-miR-30a-5p and DNMBP-AS1 are involved in various tumor occurrences and development. Hsa-miR-30a-5p functions as a tumor suppressor in lung squamous cell carcinoma, ovarian cancer, and prostate cancer by inhibiting cell proliferation, migration, invasion, and epithelial-mesenchymal transition [30–32]. However, hsa-miR-30a-5p exhibits a cancer-promoting effect in cholangiocarcinoma, oral cancer, and glioma, indicating it may function as either a tumor oncogene or suppressor depending on the specific cancer type [33, 34]. Sheng Gao and colleagues identified that DNMBP-AS low expression in breast cancer was associated with poor prognosis [35]. Moreover, DNMBP-AS1 inhibited colon cancer progression through the miR-93-5p/17-5p/NHLRC3 axis [36]. Consistent with these previous studies, we found that DNMBP-AS1 was significantly downregulated in CRC and was associated with poor prognosis. Insufficient information exists regarding the functions of DNMBP-AS1 and hsa-miR-30a-5p in the metabolism reprogramming in cancer cells. This study shows that the DNMBP-AS1/hsa-miR-30a-5p axis modulates PGC1α expression, regulating the expression of downstream critical glycolytic enzyme PKM2 and affecting the Warburg effect in CRC cells.

The role of PGC1α in cancer exhibits striking tissue specificity, reflecting its ability to engage distinct transcriptional programs across malignancies. While our study supports its tumor-suppressive function in CRC through PPARγ-mediated inhibition of Wnt/β-catenin signaling and suppression of PKM2-driven glycolysis, contrasting roles have been reported in other cancers. In prostate cancer, PGC1α activation induces an ERRα-dependent catabolic state to suppress metastasis [23], whereas in breast cancer, it enhances mitochondrial flexibility to promote metastatic dissemination [21]. The observed CRC-specific tumor suppression likely stems from PGC1α’s preferential interaction with PPARγ-a key transcriptional regulator in intestinal homeostasis-rather than alternative partners such as ERRα [29]. Despite reports suggesting a pro-tumorigenic role for elevated PGC1α in subsets of CRC [37], our clinicopathological analyses of 121 paired samples demonstrate that reduced PGC1α expression correlates with advanced tumor stages and poor prognosis. This apparent discrepancy may reflect microenvironmental differences, as murine models of intestinal dysplasia and human epidemiological studies link PGC1α upregulation through physical activity to CRC risk reduction [38, 39]. Collectively, these findings highlight how tissue-specific transcriptional partnerships, coupled with microenvironmental and metabolic constraints, dictate PGC1α’s functional duality in carcinogenesis.

In the primary and pivotal step of glycolysis, pyruvate kinase M2 (PKM2) serves as the catalyst, facilitating the conversion of phosphoenolpyruvate (PEP) to pyruvate, a crucial event in the glycolytic pathway. PKM2 holds a central role as the enzyme overseeing this irreversible and rate-limiting process, marking it as the key regulatory entity in glycolysis [40]. PKM1 and PKM2 are alternatively spliced isoforms derived from the PKM gene, encoding distinct protein products with varied functional properties. While PKM1 is constitutively active and associated with oxidative phosphorylation, PKM2, known for its regulatory flexibility, predominates in glycolytic tissues, including cancer cells. The switch between these isoforms provides cancer cells with a unique metabolic advantage, adapting to dynamic microenvironmental conditions [41]. The expression patterns of PKM2 in cancer exhibit remarkable diversity across tumor types. Notably, in esophageal cancer, elevated PKM2 expression is associated with advanced stages and poorer prognosis [42]. Lung cancer studies reveal intricate correlations, with PKM2 implicated in promoting tumor growth and metastasis [43]. Additionally, in liver cancers, PKM2 overexpression correlates with aggressive phenotypes, emphasizing its significance in hepatocellular carcinoma progression [44]. Furthermore, PKM2’s role in CRC is multifaceted. Our recent investigations indicate that PKM2 is intricately regulated by the transcriptional coactivator PGC1α, influencing glycolytic pathways and Wnt/β-catenin signaling in CRC cells. These findings align with broader literature on the interplay between Wnt pathway activation and glycolytic enzyme expression in tumor cells.

Rosiglitazone, a thiazolidinedione hypoglycemic agent, is utilized to treat type 2 diabetes. Its primary mechanism of action involves regulating glucose and lipid metabolism by binding to PPARγ [45]. The signaling of PPARγ has been linked to various diseases, including cancer. Research conducted on human tumor tissue has supported that PPARγ expression has a protective effect on CRC, and patients with higher PPARγ expression typically have a better prognosis [46, 47]. Conversely, decreased PPARγ expression has been associated with increased tumor-associated macrophages, higher metastasis rates, and reduced survival rates [48]. Herein, we measured the rosiglitazone IC50 for suppressing cell viability in diverse CRC cells ranging from 2.36–11.76 μM. Our findings suggest that rosiglitazone may modulate tumor cell glycolysis by inhibiting the Wnt signaling pathway in conjunction with PGC1α. Our results are consistent with prior research that has demonstrated PPARγ activation to be associated with inhibiting the WNT/β-catenin pathway in various pathophysiological conditions [49, 50].

Notably, our findings reveal PGC1α specifically inhibits PKM2 without altering LDHA or HK2 expression, distinguishing its mechanism from pan-glycolytic inhibitors. This specificity may arise from Wnt/β-catenin’s preferential regulation of PKM2, coupled with PGC1α-PPARγ‘s transcriptional selectivity. While LDHA and HK2 are critical for glycolysis, their HIF-1α/Myc-dependent regulation suggests a divergent pathway not governed by the PGC1α axis in CRC [51,52]. This targeted suppression of PKM2, a metabolic checkpoint, highlights a precision therapeutic opportunity to disrupt Warburg flux while preserving basal glycolysis essential for non-malignant cells.

Our study also highlights the immunomodulatory effects of PGC1α activation, as ZLN005 treatment increased CD8 + T cell infiltration and reduced PD-1 expression in MC38 tumors. While the exact mechanism linking these observations requires further exploration, we propose that PGC1α-driven suppression of glycolysis (via PKM2 downregulation) may attenuate lactate-mediated immunosuppression and hypoxia-driven PD-L1/PD-1 axis activation [53, 54]. Future studies should investigate whether PGC1α activation synergizes with PD-1 blockade by directly reprogramming T cell metabolism or indirectly remodeling the TME.”

Several limitations warrant consideration. First, while xenograft models demonstrated ZLN005’s synergy with anti-PD-1, they lack the immune heterogeneity of human CRC. Syngeneic models or humanized mice could better mimic the tumor microenvironment [55]. Second, though our clinical cohort (*n* = 121) met statistical thresholds for survival analysis, validation in larger multi-center cohorts is warranted. Despite these limitations, our findings provide mechanistic insights into PGC1α’s metabolic regulation in CRC and highlight its therapeutic potential

## Conclusion

Our results elucidate the roles and mechanisms underlying the DNMBP-AS1/hsa-miR-30a-5p/PGC1α ceRNA network in CRC proliferation, metabolic reprogramming, and immune responses. PGC1α suppresses CRC proliferation both in vitro and in vivo by inhibiting PKM2, a key enzyme in the glycolytic pathway, thereby reducing CRC aerobic glycolysis. We also demonstrated that the PGC1α agonist ZLN005 and the PPARγ agonist rosiglitazone act synergistically to inhibit CRC proliferation. Moreover, we validated that ZLN005 markedly enhances PD-1-induced tumor suppression in xenograft mice. Furthermore, the expression patterns of PGC1α and PKM are closely associated with patient prognosis. Our findings revealed the molecular mechanisms by which PGC1α inhibits CRC that enable the development of targeted drug therapies against CRC and its downstream targets.

## Materials and methods

### Microarray data

This study used two mRNA microarray datasets (GSE21815 and GSE21510) from the GEO database (http://www.ncbi.nlm.nih.gov/geo). The GSE21815 dataset included 132 CRC tissue samples and 9 para-carcinoma colorectum tissue samples, while the GSE21510 dataset comprised 123 CRC tissue samples and 25 para-carcinoma colorectum tissue samples. For additional data, we accessed RNA sequence data (lncRNA and mRNA, level 3; Illumina HiSeq RNA-Seq platform), miRNA sequence data (Illumina HiSeq miRNA-Seq platform), and relevant clinical information of CRC patients from the TCGA database. For data retrieval, we used the “TCGAbiolinks” package in the R programming language (version 4.1.4).

### Bioinformatics analysis

To identify differentially expressed genes (DEGs), miRNAs (DEmiRNAs), and lncRNAs (DElncRNAs) in colorectal cancer (CRC), we utilized the ‘limma’ package with adj. *p* < 0.05 and |log2(fold change)| > 1.5 for DEGs and DEmiRNAs, and adj. *p* < 0.05 and |log2(fold change)| > 1 for DElncRNAs. Volcano plots were generated using ggplot2 in R based on the identified genes. Protein-protein interaction (PPI) networks for upregulated and downregulated DEGs were constructed via the STRING database (http://string-db.org) with a confidence score threshold (*R* > 0.4). Hub genes were identified using the CytoHubba plugin in Cytoscape, employing the degree algorithm to select the top 20 hub genes.

Co-expression correlations between mRNA-miRNA, mRNA-lncRNA, and miRNA-lncRNA pairs in CRC samples were assessed using R, calculating correlation coefficients with TCGA data (|R| > 0.2, adj. *p* < 0.05). Results were visualized using the ‘ggscatterstats’ package. Clinical data from TCGA-COAD/READ datasets were integrated, and survival analysis was performed using the ‘survival’ and ‘survminer’ packages. Kaplan–Meier survival curves with log-rank test p-values were generated.

Comprehensive analysis of DEGs, DEmiRNAs, and DElncRNAs was conducted in TCGA-COAD/READ. miRDB, starBase, and miRNet2.0 were employed for predicting miRNA-mRNA and miRNA-lncRNA interactions. A ceRNA network was constructed based on negative miRNA-target correlation and positive mRNA-lncRNA correlation. Dual-luciferase experiments validated our findings, providing a comprehensive understanding of CRC regulatory networks and novel insights into disease mechanisms.

### Patients and specimens

This study obtained human CRC tissues and paired para-carcinoma colorectum tissues from patients at Huashan Hospital, Fudan University, Shanghai, China, between 2017 and 2022. The para-carcinoma colorectum tissues were obtained from a region at least 1 cm away from the tumor border and confirmed through microscopy to be devoid of tumor cells. The real-time polymerase chain reaction (PCR), western blotting, and immunohistochemistry analyses were used to evaluate the PGC1α expression levels in tumor versus para-carcinoma tissues and perform prognosis analysis. The Huashan Hospital Ethics Committee approved this study, and written informed written consent was obtained from all participating patients.

### Cell culture

The human CRC cell lines SW480, SW620, HCT116, HT29, LoVo, NCM460 and mouse colorectal cancer cell line MC38 were obtained from the Cell Bank Type Culture Collection of the Chinese Academy of Sciences (Shanghai, China). HT29, NCM460 and MC38 were cultured in Roswell Park Memorial Institute-1640 medium (RPMI-1640, Hyclone, USA). HCT116 was cultured in 50% McCoy’s 5a medium (Hyclone) and 50% RPMI-1640 mixed medium. SW480 and SW460 were cultured in Leibovitz’s L-15 medium (L15, Hyclone). LOVO cultured in Ham’s F-12K medium (F12K, Hyclone). Both culture media were supplemented with 10% fetal bovine serum (FBS, Yeasen Biotechnology Co., Ltd, China) and 100 U/mL penicillin/streptomycin (Wisent). The cultures were maintained in a humidified CO_2_ incubator (5% CO_2_; 95% air; 37 °C). The vendors of the cells verified and described each one.

### Reagents

Rosiglitazone (selective PPARγ agonist, HY-17386), ZLN005 (selective PGC1α agonist, HY-17538), oligomycin (HY-N6782), carbonyl cyanide 4-(trifluoromethoxy) phenylhydrazone (HY-100410), glucose (HY-B0389) and 2-deoxyglucose (HY-13966) were purchased from Med Chem Express (USA) and dissolved in dimethyl sulfoxide (DMSO). These reagents were stored at –20 °C.

### Virus production and small interfering RNA

The lentiviral expression system was utilized to investigate PGC1α overexpression or knockdown. Lentiviral vectors of PGC1α and negative control were obtained from GeneChem Co (Shanghai, China). The CRC cells (6 × 10^5^) were seeded on six-well plates and infected with the PGC1α or negative control lentiviral vector in 2 mL of culture medium at an infection multiplicity of 35 plaque-forming units/cell, followed by adding ten times the volume of virus-enhanced infection solution. After 8-h incubation, the medium was replaced with 2 mL of fresh medium containing 10% FBS and 1% antibiotic-antimycotic. Stable PGC1α-overexpressing or knockdown cell lines were selected with 1 μg/mL puromycin (Invitrogen, A1113802). The cells were trypsinized after 72 h and prepared for Western blot or qRT-PCR analysis.

### Cell viability assay

The Cell Counting Kit-8 (Proteintech Group, Inc, USA) was employed to perform a cell viability assay. Specifically, CRC cells were seeded into 96-well plates at a concentration of 6 × 10^3^ cells/well and incubated under standard culture conditions for 24 h. A 10 µL of CCK-8 solution was added to each well and incubated for 1 h at 37 °C per the protocols. A microplate reader (Biotek, Winooski, VT, USA) was utilized to measure the optical density (OD) values at 450 nm. Each experiment was conducted independently three times, with five replicates each.

### Colony formation assay

A colony formation assay was used to evaluate cell proliferation. Specifically, CRC cells were plated in 6-well plates at 1 × 10^3^ cells/well and cultured under standard conditions for seven days. The cells were then fixed with 4% paraformaldehyde and stained with 0.1% crystal violet for 30 min. The stained colonies were photographed using a microscope, using Image J software to quantify the number of clones.

### EdU assay

To evaluate DNA synthesis, the EdU assay was conducted on CRC cells seeded in 6-well plates (1 × 10^6^ cells/well) and cultivated overnight. The cells were then incubated with 50 μmol/L EdU (Servicebio, Wuhan, China) for 2 h and stained following the protocols. Images were captured using a fluorescence microscope (Nikon, Japan), and the percentage of EdU-positive cells was calculated from five random fields in three wells using ImageJ software. DAPI was used to label the nucleus, which appears blue.

### Assays of ECAR and OCR

Briefly, cells were seeded in 96-well plates at 10,000 cells/well and treated with extracellular acidification rate (ECAR) reagents following the manufacturer (ab197244, Abcam, UK). Micro-plate readers (Victor, 3 PerkinElmer) collected ECAR signals every 5 min for 120 min using 380 nm excitation and 615 nm emission wavelengths, respectively. Then, the oxygen consumption rate (OCR) was measured using a Seahorse XF24 Analyzer (Seahorse). An equilibration medium containing 25 mM glucose, 1 mM pyruvate, and 2 mM glutamine was used to equilibrate cells in a bicarbonate-free medium. All measurements were performed in three wells per condition per experiment and repeated at least three times [56].

### Measurement of Glucose Uptake, Lactate Production and ATP Content

CRC cells (1 × 10^6^) were seeded into a 6-well plate and cultured for 24 h until cells adhered. ATP levels were assessed per the ATP Assay kit instructions (Sangon Biotech, Shanghai, China). To measure glucose and lactate concentrations, the culture medium was swapped with phenol-red free DMEM containing 1% FBS for another 24 h, after which the medium was collected. Glucose uptake was evaluated using a kit (Abcam, USA), while the consumed glucose level was determined by subtracting the detected glucose concentration in the medium from the initial one. The lactate production assessment followed the Lactate Assay kit instructions (Sangon Biotech, Shanghai, China). The obtained results were adjusted based on cell numbers to ensure accuracy.

### Western blot analysis

Western blotting was performed as described previously. The primary antibodies used were β-catenin (#8480), c-Myc (#18583), PKM2 (#3198), PGC1α (#2178), LDHA (#3582), HK2 (#55205) (Cell Signaling Technology), and β-actin (sc-8432) (Santa Cruz Biotechnology). These primary antibodies were followed by appropriate secondary antibodies conjugated to horseradish peroxidase. Antibody-protein complexes were detected using enhanced chemiluminescence (ECL) immunoblotting detection reagent. The signals were analyzed using a LAS-3000 image analyzer and MultiGauge software (Fuji Film).

### Quantitative real‑time polymerase chain reaction

Total RNA was isolated using the Trizol reagent and Ultrapure RNA kit (CW Biotech, China) according to the manufacturer’s instructions. A 2 mg of total mRNA was reverse-transcribed into cDNA using the Superscript™ reverse transcription system (Takara, Dalian, Japan). Quantitative real-time reverse transcription polymerase chain reaction (RT-PCR) reactions were performed on an ABI 7500 Real-Time PCR system (Applied Biosystems, Foster City, CA, USA) using SYBR Green PCR master mix reagents (Takara, Dalian, Japan). Relative quantification of the target gene was calculated after being normalized to GAPDH gene expression using the 2^−△△Ct^ method. The primer sequences were selected based on the RTPrimerDB database (http://medgen.ugent.be/rtprimerdb/).

### CD8^+^ T cell culture and CD8^+^ T cell-mediated colorectal cancer cell killing assay

Peripheral blood mononuclear cells (PBMCs) from a healthy individual were isolated using PBMC separation reagent (Yeasen Biotechnology Co., Ltd, China) and were then cultured in RPMI-1640 medium. CD8 T cells, attached to CD8 microbeads (Invitrogen, USA), were separated from PBMCs through magnetic separation. Flow cytometry, utilizing FITC-CD3 antibodies (Abcam, UK) and APC-CD8 antibodies (Abcam, UK), determined the positive rate of cell sorting. CD8^+^ T cells, cultured in RPMI-1640 medium, were activated by introducing CD3 antibodies (2 μg/mL; Invitrogen, USA), CD28 antibodies (1 μg/mL; Invitrogen, USA), and interleukin 2 (IL-2, 5 ng/mL; Yeasen Biotechnology Co., Ltd, China). Following 72 h, these activated CD8^+^ T cells were co-cultured with colorectal cancer cells at a ratio of 1:2.5 for 48 h. Subsequently, an appropriate amount of medium was collected from the co-culture system to assess granzyme B and IFN-γ content using an ELISA kit (Sangon Biotech, China). The cytotoxicity was examined by co-culturing activated CD8^+^ T cells with colorectal cancer cells at a ratio of 2:1 for 72 h. After eliminating debris and T cells through multiple PBS washes, the killing ability of CD8^+^ T cells on colorectal cancer cells was evaluated using CCK8 and flow apoptosis assays.

### RNA-Seq and data analysis

Total RNA was extracted from HCT116 cells with PGC1α overexpression and control cells using Trizol reagent (Invitrogen, Carlsbad) following the protocol. The quality and quantity of RNA were assessed using a NanoDrop ND-1000 spectrophotometer. The cDNA library was prepared using the MGI Stranded RNA-Seq Library Preparation Kit per the manufacturer’s instructions. RNA sequencing was performed on an MGI MGISEQ-2000 system following protocols. To gain further insight into the biological pathways involved in CRC related to PGC1α, gene set enrichment analysis (GSEA) was conducted.

### Dual-luciferase assays

Transcriptional regulation studies employed dual-luciferase reporter systems to investigate miRNA-mRNA interactions and signaling pathway activity. For miRNA target validation, wild-type and mutagenized 3ʹUTR sequences of PGC1α and DNMBP-AS1 containing predicted miR-30a-5p binding sites were synthesized and cloned into the psiCHECK-2 dual-reporter vector. SW620 and HT29 colon cancer cells were co-transfected with 400 ng reporter constructs and 50 nM miR-30a-5p mimic or negative control RNA using Lipofectamine 2000 (Invitrogen). Wnt/β-catenin pathway activity was concurrently assessed using the TOPFlash TCF/LEF-responsive firefly luciferase system (Beyotime), with HCT116 cells co-transfected with 200 ng TOPFlash plasmid and 10 ng pRL-TK Renilla luciferase control using Lipofectamine 2000 (Invitrogen). For mechanistic studies, Wnt reporter assays incorporated co-transfection with lentiviral vectors of PGC1α overexpression or empty vector, with subsets of cultures pretreated with 10 µM GW9662 PPARγ inhibitor 24 h post-transfection.

All transfections were normalized to Renilla luciferase activity using the Dual-Luciferase Reporter Assay System (Promega), with measurements performed 48 hr after transfection on a SpectraMax M5 microplate reader. Firefly luciferase signals were quantified relative to Renilla values for pathway activity assessment, while psiCHECK-2 experiments calculated the ratio of Renilla (target reporter) to firefly (constitutive control) luminescence. Experiments included three biological replicates with triplicate technical measurements per condition, with normalization performed against vector-transfected controls.

### Co-immunoprecipitation assays

Protein-protein interactions between PGC1α and PPARγ were investigated in HCT116 cells using co-immunoprecipitation under optimized experimental conditions. Cultured cells were lysed in RIPA buffer supplemented with protease and phosphatase inhibitors. Lysates were pre-cleared with protein A/G agarose beads (Santa Cruz Biotechnology, sc-2003) for 1 h at 4 °C to minimize non-specific binding. For immunoprecipitation, 500 µg of total protein lysate was incubated overnight at 4 °C with 2 µg of anti- PPARγ monoclonal antibody (Abcam, ab45036) or species-matched IgG control (Abcam, ab316981). Immune complexes were captured using protein A/G magnetic beads (Pierce) with continuous rotation for 2 h, followed by three washes with ice-cold lysis buffer containing 150 mM NaCl. Bound proteins were eluted in Laemmli buffer, resolved by SDS-PAGE, and immunoblotted with anti-PPARγ (Abcam, ab316981) antibody and anti-PGC1α to confirm interaction specificity.

### Histopathology and IHC

To evaluate PGC1α expression in patient tissue sections, the sections were subjected to antigen retrieval by heating in a microwave oven at 100 °C for 15 min using citrate buffer (pH 6.0) after deparaffinization and rehydration. The sections were incubated with a PGC1α antibody (1:200) overnight at 4 °C. Subsequently, a secondary antibody (goat anti-rabbit conjugated to horseradish peroxidase, 1:200; #ab97051, Abcam) was applied for 1 h at room temperature. The sections were stained using 3,3′-diaminobenzidine tetrahydrochloride (Long Island, Shanghai, China), and PGC1α expression was assessed based on staining intensity and extent.

### Tumor immune phenotyping and flow cytometry analysis

Tumors were procured and mechanically dissociated into fragments within a span of 2 h. Subsequently, the tumor tissue underwent disruption to yield single cells, utilizing a tumor isolation kit (Miltenyi Biotec) in accordance with the provided guidelines. The resultant cell suspension underwent lysis employing a 70 mm cell filter to eliminate red blood cells. Tumor-infiltrating leukocytes were segregated through gradient centrifugation using a 40%/80% Percoll (GE Healthcare) solution. Following this, the amassed cells were subjected to blocking with Fc block (anti-mouse CD16/32, BioLegend) on ice for a duration of 30 min.

The samples underwent initial staining for surface markers pertaining to lymphoid immune populations, succeeded by intracellular staining. For FoxP3 and intracellular staining, the True-Nuclear Transcription Factor Buffer Set 424401 (BioLegend) was employed in adherence to the manufacturer’s instructions. The antibodies and stain kit, namely APC-Cy7 anti-mouse CD45, Alexa Fluor 700 Hamster anti-mouse CD3e, FITC anti-mouse CD4, PerCP-Cy5.5 anti-mouse CD8a, BV786 anti-mouse CD45R/B220, PE anti-mouse NK-1.1, BV650 anti-mouse IFN-γ, Alexa Fluor 647 anti-mouse Foxp3, and fixable viability stain 510, were procured from BioLegend. Additionally, PE-CYN7 anti-mouse granzyme B was sourced from Thermo Fisher Scientific. The gating strategy for flow cytometry analysis of lymphoid and myeloid populations in MC38 tumors is shown in Fig. S5.

### CRC xenografts

Human CRC cell xenografts were established by subcutaneously injecting 2 × 10^6^ CRC cells suspended in PBS/Matrigel into the right flank region of 8-week-old male BALB/c nude mice. Tumor volume was monitored every 5 days after reaching 100 mm³ using caliper measurements (volume = length × width² × 0.5). This interval was selected to minimize animal stress while ensuring sufficient temporal resolution for growth rate calculations, consistent with prior studies [57, 58]. No protocol violations or missed time points occurred during the study.

In the pharmacological intervention experiments, we intragastrically injected ZLN005 (20 mg/kg), rosiglitazone (100 mg/kg), ZLN005 (20 mg/kg) + rosiglitazone (100 mg/kg), or vehicle (1% DMSO + ddH_2_O) every two days for a total of 25 days, starting from day 5 after tumor injection. For immune checkpoint inhibitor synergy studies, MC38 colorectal cancer cells were subcutaneously injected (1 × 10^6^ cells/mouse) into immunocompetent 8-week-old male C57BL/6J mice to establish syngeneic tumors. Beginning on day 5 post-injection, mice were intraperitoneally treated with ZLN005 (20 mg/kg) or vehicle every 2 days for 3 weeks, combined with anti-PD-1 antibodies (100 µg/mouse, BioXCell) or IgG isotype control administered intraperitoneally every 3 days. Our animal procedures were approved by Fudan University’s Institutional Animal Care and Use Committee, ensuring to minimize discomfort and distress to the animals throughout the study.

### Machine learning feature assessment

Selected features were assessed using six machine-learning algorithms: Random Forests (RF), Support Vector Machines (SVM), eXtreme Gradient Boosting (XGB), K Nearest Neighbors (KNN), Naive Bayes (NB) and Linear Discriminant Analysis (LDA). The prediction was performed using mlr3 package (version 0.16.1) from R language. To evaluate the performance of selected features in TCGA-COAD cohort (*n* = 156), 10-fold cross validation generated six models and the area under the curve (AUC) for the receiver operating characteristic (ROC) was obtained.

### Statistical analysis

Graph Pad Prism 7.0 and SPSS 16.0 software were used for statistical evaluation. Group differences in vitro experiments were compared by performing a one-way analysis of variance (ANOVA) with Dunnett’s post hoc test. The animal experiments were compared using independent-sample Student t-tests. Tumor-bearing mice were allocated to treatment groups through computer-generated random sequences (http://www.randomizer.org) after reaching 100 mm³ tumor volume. Investigators were blinded to group allocation during drug administration and outcome assessment. Before analysis, all data underwent the Kolmogorov–Smirnov normality test and Levene’s test; results showed that all data from different data groups satisfied the normality and homogeneity of variance. Data are expressed as mean ± SD. *P* < 0.05 was considered statistically significant. All authors had access to the study data and had reviewed and approved the final manuscript.

Random allocation protocols (Materials and Methods/CRC xenografts): Animals were randomized to treatment groups using a computer-generated sequence (block randomization, block size = 10) post-tumor inoculation (100 mm³), with randomization codes maintained by an independent technician. Cells for transplantation were pretested for mycoplasma contamination and allocated blindly.

Blinding procedures: Outcome assessments (tumor volume measurements, histopathological scoring) were performed by two investigators masked to group assignments. Blinding was maintained until statistical analyses were completed.

## Supplementary information

supplementary materials

Supplementary Code File 1

Supplementary Data File 1

supplementary results

## Data Availability

The RNA-sequencing datasets generated during this study are available in Supplementary Data File 1. The machine learning code and model parameters are provided in Supplementary Code File 1. And all other the raw data available from the corresponding author on reasonable request.

## References

[1] Fleming M, Ravula S, Tatishchev SF, Wang HL. Colorectal carcinoma: pathologic aspects. J Gastrointest Oncol. 2012;3:153–73. 22943008 10.3978/j.issn.2078-6891.2012.030 PMC3418538

[2] Bray F, Ferlay J, Soerjomataram I, Siegel RL, Torre LA, Jemal A. Global cancer statistics 2018: GLOBOCAN estimates of incidence and mortality worldwide for 36 cancers in 185 countries. CA Cancer J Clin. 2018;68:394–424. 30207593 10.3322/caac.21492

[3] Kuipers EJ, Grady WM, Lieberman D, Seufferlein T, Sung JJ, Boelens PG, et al. Colorectal cancer. Nat Rev Dis Prim. 2015;1:15065. 27189416 10.1038/nrdp.2015.65 PMC4874655

[4] O’Connell JB, Maggard MA, Ko CY. Colon cancer survival rates with the new American Joint Committee on Cancer sixth edition staging. J Natl Cancer Inst. 2004;96:1420–5. 15467030 10.1093/jnci/djh275

[5] Davila RE, Rajan E, Baron TH, Adler DG, Egan JV, Faigel DO, et al. ASGE guideline: colorectal cancer screening and surveillance. Gastrointest Endosc. 2006;63:546–57. 16564851 10.1016/j.gie.2006.02.002

[6] Hanahan D, Weinberg RA. Hallmarks of cancer: the next generation. Cell. 2011;144:646–74. 21376230 10.1016/j.cell.2011.02.013

[7] Koppenol WH, Bounds PL, Dang CV. Otto Warburg’s contributions to current concepts of cancer metabolism. Nat Rev Cancer. 2011;11:325–37. 21508971 10.1038/nrc3038

[8] Hsu PP, Sabatini DM. Cancer cell metabolism: Warburg and beyond. Cell. 2008;134:703–7. 18775299 10.1016/j.cell.2008.08.021

[9] Salmena L, Poliseno L, Tay Y, Kats L, Pandolfi PP. A ceRNA hypothesis: the Rosetta Stone of a hidden RNA language?. Cell. 2011;146:353–8. 21802130 10.1016/j.cell.2011.07.014 PMC3235919

[10] Hua Q, Jin M, Mi B, Xu F, Li T, Zhao L, et al. LINC01123, a c-Myc-activated long non-coding RNA, promotes proliferation and aerobic glycolysis of non-small cell lung cancer through miR-199a-5p/c-Myc axis. J Hematol Oncol. 2019;12:91. 31488218 10.1186/s13045-019-0773-y PMC6728969

[11] Liu X, Zhu Q, Guo Y, Xiao Z, Hu L, Xu Q. LncRNA LINC00689 promotes the growth, metastasis and glycolysis of glioma cells by targeting miR-338-3p/PKM2 axis. Biomed Pharmacother. 2019;117:109069. 31181442 10.1016/j.biopha.2019.109069

[12] Chen J, Yu Y, Li H, Hu Q, Chen X, He Y, et al. Long non-coding RNA PVT1 promotes tumor progression by regulating the miR-143/HK2 axis in gallbladder cancer. Mol Cancer. 2019;18:33. 30825877 10.1186/s12943-019-0947-9 PMC6397746

[13] Shen Y, Xu J, Pan X, Zhang Y, Weng Y, Zhou D, et al. LncRNA KCNQ1OT1 sponges miR-34c-5p to promote osteosarcoma growth via ALDOA enhanced aerobic glycolysis. Cell Death Dis. 2020;11:278. 32332718 10.1038/s41419-020-2485-1 PMC7181648

[14] D'errico I, Salvatore L, Murzilli S, Lo Sasso G, Latorre D, Martelli N, et al. Peroxisome proliferator-activated receptor-gamma coactivator 1-alpha (PGC1alpha) is a metabolic regulator of intestinal epithelial cell fate. Proc Natl Acad Sci USA. 2011;108:6603–8. 21467224 10.1073/pnas.1016354108 PMC3081029

[15] Villena JA. New insights into PGC-1 coactivators: redefining their role in the regulation of mitochondrial function and beyond. FEBS J. 2015;282:647–72. 25495651 10.1111/febs.13175

[16] Stupp R, Mason WP, van den Bent MJ, Weller M, Fisher B, Taphoorn MJ, et al. Radiotherapy plus concomitant and adjuvant temozolomide for glioblastoma. N Engl J Med. 2005;352:987–96. 15758009 10.1056/NEJMoa043330

[17] Mastropasqua F, Girolimetti G, Shoshan M. PGC1alpha: friend or foe in cancer?. Genes. 2018;9:48. 29361779 10.3390/genes9010048 PMC5793199

[18] LaGory EL, Wu C, Taniguchi CM, Ding CC, Chi JT, von Eyben R, et al. Suppression of PGC-1alpha is critical for reprogramming oxidative metabolism in renal cell carcinoma. Cell Rep. 2015;12:116–27. 26119730 10.1016/j.celrep.2015.06.006 PMC4518559

[19] Tennakoon JB, Shi Y, Han JJ, Tsouko E, White MA, Burns AR, et al. Androgens regulate prostate cancer cell growth via an AMPK-PGC-1alpha-mediated metabolic switch. Oncogene. 2014;33:5251–61. 24186207 10.1038/onc.2013.463 PMC4009392

[20] Luo C, Lim JH, Lee Y, Granter SR, Thomas A, Vazquez F, et al. A PGC1alpha-mediated transcriptional axis suppresses melanoma metastasis. Nature. 2016;537:422–6. 27580028 10.1038/nature19347 PMC5161587

[21] Andrzejewski S, Klimcakova E, Johnson RM, Tabariès S, Annis MG, McGuirk S, et al. PGC-1alpha promotes breast cancer metastasis and confers bioenergetic flexibility against metabolic drugs. Cell Metab. 2017;26:778–787.e5. 28988825 10.1016/j.cmet.2017.09.006

[22] Yang H, Yang R, Liu H, Ren Z, Kong F, Li D, et al. Synergism between PGC-1alpha and estrogen in the survival of endometrial cancer cells via the mitochondrial pathway. Onco Targets Ther. 2016;9:3963–73. 27418839 10.2147/OTT.S103482 PMC4935004

[23] Torrano V, Valcarcel-Jimenez L, Cortazar AR, Liu X, Urosevic J, Castillo-Martin M, et al. The metabolic co-regulator PGC1alpha suppresses prostate cancer metastasis. Nat Cell Biol. 2016;18:645–56. 27214280 10.1038/ncb3357 PMC4884178

[24] Feilchenfeldt J, Bründler MA, Soravia C, Tötsch M, Meier CA. Peroxisome proliferator-activated receptors (PPARs) and associated transcription factors in colon cancer: reduced expression of PPARgamma-coactivator 1 (PGC-1). Cancer Lett. 2004;203:25–33. 14670614 10.1016/j.canlet.2003.08.024

[25] Monami M, Lamanna C, Marchionni N, Mannucci E. Rosiglitazone and risk of cancer: a meta-analysis of randomized clinical trials. Diab Care. 2008;31:1455–60. 10.2337/dc07-2308 PMC2453648 18375416

[26] Chang CH, Lin JW, Wu LC, Lai MS, Chuang LM, Chan KA. Association of thiazolidinediones with liver cancer and colorectal cancer in type 2 diabetes mellitus. Hepatology. 2012;55:1462–72. 22135104 10.1002/hep.25509

[27] Zuo Q, He J, Zhang S, Wang H, Jin G, Jin H, et al. PPARgamma coactivator-1alpha suppresses metastasis of hepatocellular carcinoma by inhibiting Warburg effect by PPARgamma-dependent WNT/beta-catenin/pyruvate dehydrogenase kinase isozyme 1 axis. Hepatology. 2021;73:644–60. 32298475 10.1002/hep.31280

[28] Nimmakayala RK, Rauth S, Chirravuri Venkata R, Marimuthu S, Nallasamy P, Vengoji R, et al. PGC1alpha-mediated metabolic reprogramming drives the stemness of pancreatic precursor lesions. Clin Cancer Res. 2021;27:5415–29. 34172498 10.1158/1078-0432.CCR-20-5020 PMC8709878

[29] Bost F, Kaminski L. The metabolic modulator PGC-1alpha in cancer. Am J Cancer Res. 2019;9:198–211. 30906622 PMC6405967

[30] Yang J, Rao S, Cao R, Xiao S, Cui X, Ye L. miR-30a-5p suppresses lung squamous cell carcinoma via ATG5 - mediated autophagy. Aging. 2021;13:17462–72. 34253689 10.18632/aging.203235 PMC8312466

[31] Wang L, Zhao S, Yu M. Mechanism of low expression of miR-30a-5p on epithelial-mesenchymal transition and metastasis in ovarian cancer. DNA Cell Biol. 2019;38:341–51. 30839226 10.1089/dna.2018.4396

[32] Zhao H, Lai X, Zhang W, Zhu H, Zhang S, Wu W, et al. MiR-30a-5p frequently downregulated in prostate cancer inhibits cell proliferation via targeting PCLAF. Artif Cells Nanomed Biotechnol. 2019;47:278–89. 30669858 10.1080/21691401.2018.1553783

[33] Zhang JW, Wang X, Li GC, Wang D, Han S, Zhang YD, et al. MiR-30a-5p promotes cholangiocarcinoma cell proliferation through targeting SOCS3. J Cancer. 2020;11:3604–14. 32284757 10.7150/jca.41437 PMC7150463

[34] Zhao P, Wang M, An J, Sun H, Li T, Li D. A positive feedback loop of miR-30a-5p-WWP1-NF-kappaB in the regulation of glioma development. Int J Biochem Cell Biol. 2019;112:39–49. 30978403 10.1016/j.biocel.2019.04.003

[35] Gao S, Lu X, Ma J, Zhou Q, Tang R, Fu Z, et al. Comprehensive analysis of lncRNA and miRNA regulatory network reveals potential prognostic non-coding RNA involved in breast cancer progression. Front Genet. 2021;12:621809. 34220926 10.3389/fgene.2021.621809 PMC8253500

[36] Yang L, Yang T, Wang H, Dou T, Fang X, Shi L, et al. DNMBP-AS1 regulates NHLRC3 expression by sponging miR-93-5p/17-5p to inhibit colon cancer progression. Front Oncol. 2022;12:765163. 35574307 10.3389/fonc.2022.765163 PMC9092830

[37] Cho JG, Park SJ, Han SH, Park JI. PGC-1alpha regulates cell proliferation, migration, and invasion by modulating leucyl-tRNA synthetase 1 expression in human colorectal cancer cells. Cancers. 2022;15:159. 36612155 10.3390/cancers15010159 PMC9818264

[38] Bhalla K, Hwang BJ, Dewi RE, Ou L, Twaddel W, Fang HB, et al. PGC1alpha promotes tumor growth by inducing gene expression programs supporting lipogenesis. Cancer Res. 2011;71:6888–98. 21914785 10.1158/0008-5472.CAN-11-1011 PMC3282487

[39] de Souza-Teixeira F, Alonso-Molero J, Ayán C, Vilorio-Marques L, Molina AJ, González-Donquiles C, et al. PGC-1alpha as a biomarker of physical activity-protective effect on colorectal cancer. Cancer Prev Res. 2018;11:523–34. 10.1158/1940-6207.CAPR-17-0329 29789344

[40] Zhu S, Guo Y, Zhang X, Liu H, Yin M, Chen X, et al. Pyruvate kinase M2 (PKM2) in cancer and cancer therapeutics. Cancer Lett. 2021;503:240–8. 33246091 10.1016/j.canlet.2020.11.018

[41] Dayton TL, Jacks T, Vander Heiden MG. PKM2, cancer metabolism, and the road ahead. EMBO Rep. 2016;17:1721–30. 27856534 10.15252/embr.201643300 PMC5283597

[42] Ma R, Liu Q, Zheng S, Liu T, Tan D, Lu X. PKM2-regulated STAT3 promotes esophageal squamous cell carcinoma progression via TGF-beta1-induced EMT. J Cell Biochem. 2019;120:11539–50. 30756445 10.1002/jcb.28434

[43] Wang C, Zhang S, Liu J, Tian Y, Ma B, Xu S, et al. Secreted pyruvate kinase M2 promotes lung cancer metastasis through activating the integrin beta1/FAK signaling pathway. Cell Rep. 2020;30:1780–1797.e6. 32049010 10.1016/j.celrep.2020.01.037

[44] Li TE, Wang S, Shen XT, Zhang Z, Chen M, Wang H, et al. PKM2 Drives Hepatocellular Carcinoma Progression by Inducing Immunosuppressive Microenvironment. Front Immunol. 2020;11:589997. 33193421 10.3389/fimmu.2020.589997 PMC7606949

[45] Montaigne D, Butruille L, Staels B. PPAR control of metabolism and cardiovascular functions. Nat Rev Cardiol. 2021;18:809–23. 34127848 10.1038/s41569-021-00569-6

[46] Dai Y, Wang W. Peroxisome proliferator-activated receptor gamma and colorectal cancer. World J Gastrointest Oncol. 2010;2:159–64. 21160824 10.4251/wjgo.v2.i3.159 PMC2999174

[47] Ogino S, Shima K, Baba Y, Nosho K, Irahara N, Kure S, et al. Colorectal cancer expression of peroxisome proliferator-activated receptor gamma (PPARG, PPARgamma) is associated with good prognosis. Gastroenterology. 2009;136:1242–50. 19186181 10.1053/j.gastro.2008.12.048 PMC2663601

[48] Pancione M, Forte N, Sabatino L, Tomaselli E, Parente D, Febbraro A, et al. Reduced beta-catenin and peroxisome proliferator-activated receptor-gamma expression levels are associated with colorectal cancer metastatic progression: correlation with tumor-associated macrophages, cyclooxygenase 2, and patient outcome. Hum Pathol. 2009;40:714–25. 19121846 10.1016/j.humpath.2008.08.019

[49] Moldes M, Zuo Y, Morrison RF, Silva D, Park BH, Liu J, et al. Peroxisome-proliferator-activated receptor gamma suppresses Wnt/beta-catenin signalling during adipogenesis. Biochem J. 2003;376:607–13. 12954078 10.1042/BJ20030426 PMC1223802

[50] Sharma C, Pradeep A, Wong L, Rana A, Rana B. Peroxisome proliferator-activated receptor gamma activation can regulate beta-catenin levels via a proteasome-mediated and adenomatous polyposis coli-independent pathway. J Biol Chem. 2004;279:35583–94. 15190077 10.1074/jbc.M403143200

[51] You Q, Wang J, Yu Y, Li F, Meng L, Chen M, et al. The histone deacetylase SIRT6 promotes glycolysis through the HIF-1alpha/HK2 signaling axis and induces erlotinib resistance in non-small cell lung cancer. Apoptosis. 2022;27:883–98. 35915188 10.1007/s10495-022-01751-y PMC9617843

[52] Guan H, Luo W, Liu Y, Li M. Novel circular RNA circSLIT2 facilitates the aerobic glycolysis of pancreatic ductal adenocarcinoma via miR-510-5p/c-Myc/LDHA axis. Cell Death Dis. 2021;12:645. 34168116 10.1038/s41419-021-03918-y PMC8225611

[53] Zhao Z, Ji M, Wang Q, He N, Li Y. miR-16-5p/PDK4-Mediated metabolic reprogramming is involved in chemoresistance of cervical cancer. Mol Ther Oncolytics. 2020;17:509–17. 32577500 10.1016/j.omto.2020.05.008 PMC7301169

[54] Zhu S, Chen C, Hao Y. LncRNA KCNQ1OT1 acts as miR-216b-5p sponge to promote colorectal cancer progression via up-regulating ZNF146. J Mol Histol. 2021;52:479–90. 33394291 10.1007/s10735-020-09942-0

[55] Deng S, Cheng D, Wang J, Gu J, Xue Y, Jiang Z, et al. MYL9 expressed in cancer-associated fibroblasts regulate the immune microenvironment of colorectal cancer and promotes tumor progression in an autocrine manner. J Exp Clin Cancer Res. 2023;42:294. 37926835 10.1186/s13046-023-02863-2 PMC10626665

[56] Feng J, Yang H, Zhang Y, Wei H, Zhu Z, Zhu B, et al. Tumor cell-derived lactate induces TAZ-dependent upregulation of PD-L1 through GPR81 in human lung cancer cells. Oncogene. 2017;36:5829–39. 28604752 10.1038/onc.2017.188

[57] Shen H, Ojo OA, Ding H, Mullen LJ, Xing C, Hossain MI, et al. HIF1alpha-regulated glycolysis promotes activation-induced cell death and IFN-gamma induction in hypoxic T cells. Nat Commun. 2024;15:9394. 39477954 10.1038/s41467-024-53593-8 PMC11526104

[58] Chen A, Neuwirth I, Herndler-Brandstetter D. Modeling the tumor microenvironment and cancer immunotherapy in next-generation humanized mice. Cancers. 2023;15:2989. 37296949 10.3390/cancers15112989 PMC10251926

