# Supplementary material for: DNMBP-AS1/hsa-miR-30a-5p/PGC1α axis suppresses tumor progression of colorectal cancer by inhibiting PKM2-mediated Warburg effect and enhance anti-PD-1 therapy efficacy
Source: Cell Death Discov. 2025 Jul 2;11:299. doi: 10.1038/s41420-025-02561-2 (PMC12222716; doi:10.1038/s41420-025-02561-2)

DNMBP-AS1/hsa-miR-30a-5p/PGC1α Axis Suppresses Tumor Progression of Colorectal Cancer by Inhibiting PKM2-mediated Warburg Effect and enhance anti-PD-1 therapy efficacy

Tianxiao Wang[#1], Wenxin Zhang [#1], Jiafeng Liu[1], Xiang Mao[2], Xinhai Wang[2], Jiyifan Li[1], Yuxin Huang[1], Zimei Wu[1], Haifei Chen[1], Huanying Shi[1], Huijie Qi[1], Lu Chen[*1], Qunyi Li [*1].

1.Department of Pharmacy, Huashan Hospital, Fudan University, Shanghai, China

2.Department of Surgery, Huashan Hospital, Fudan University, Shanghai, China

[#]These two authors contributed equally to this work.

The protein marker used in this work was EZ Mark 10-180bp DNA ladderpurchased from Shanghai LJ Biotechnology (band profile see below)

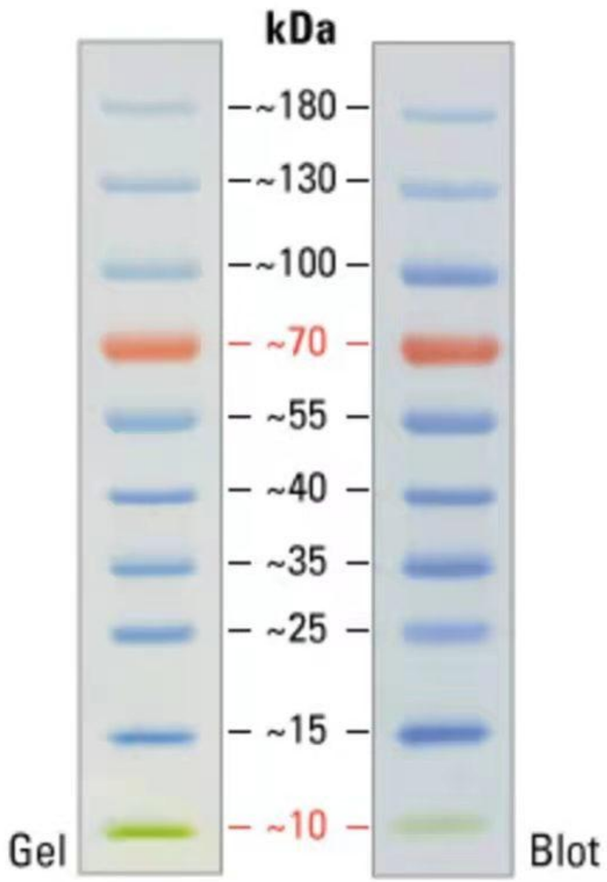

Full unedited blot for Figure 3D

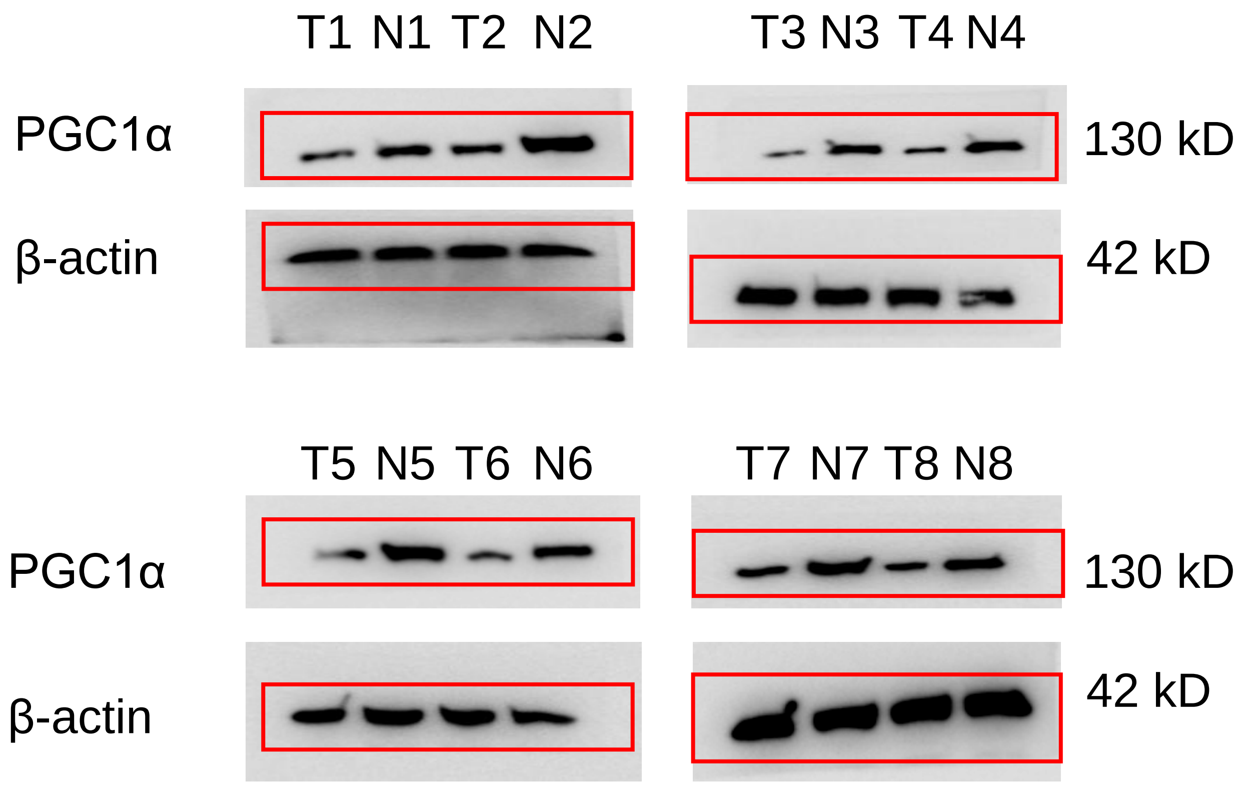

Full unedited blot for Figure 5O

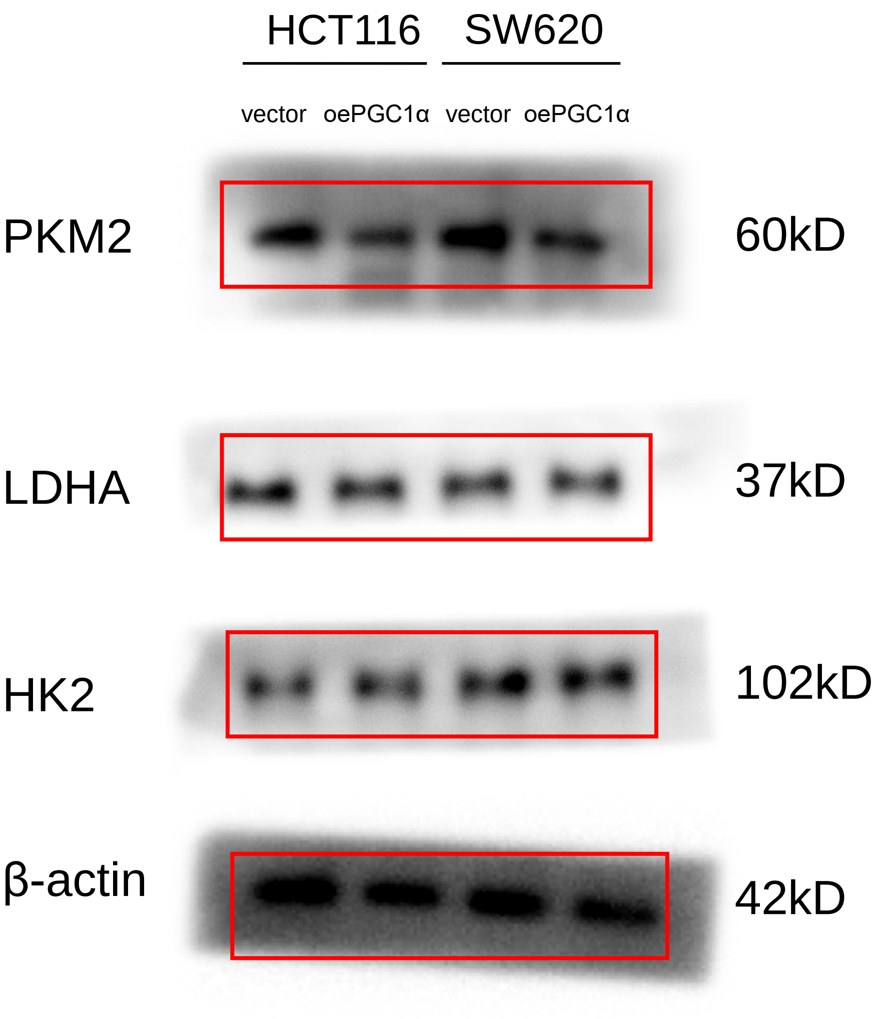

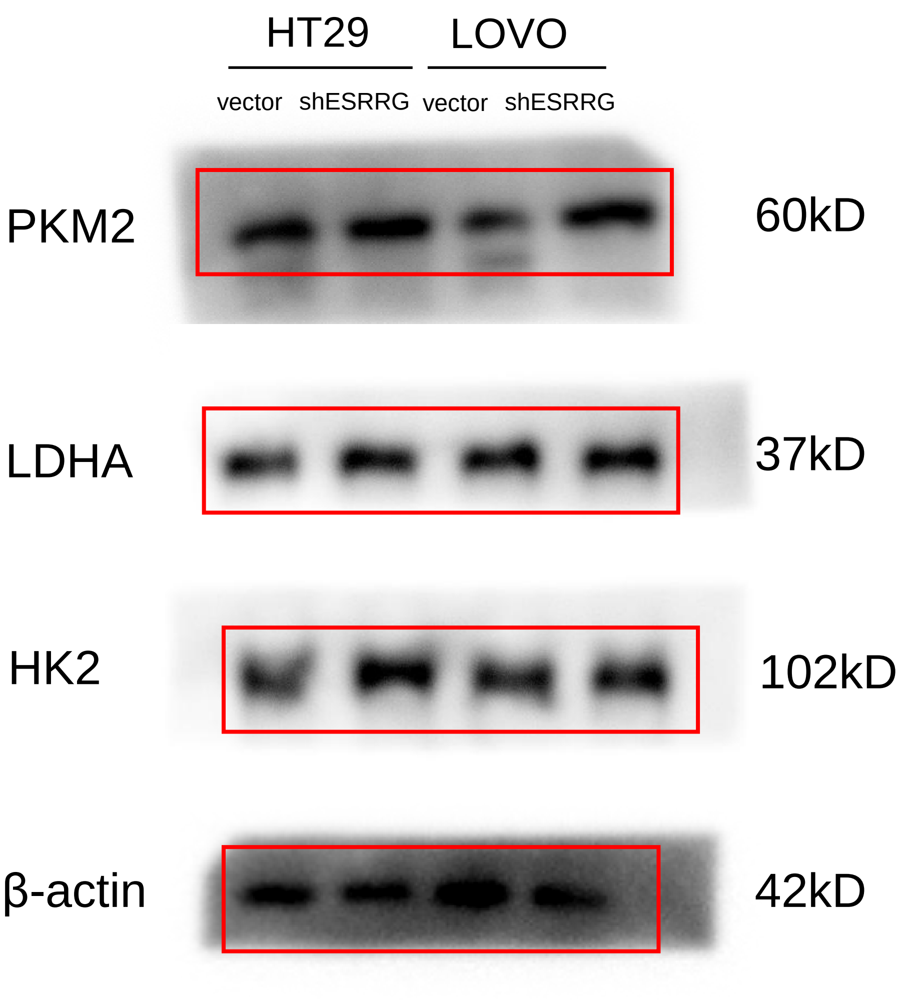

Full unedited blot for Figure 6A

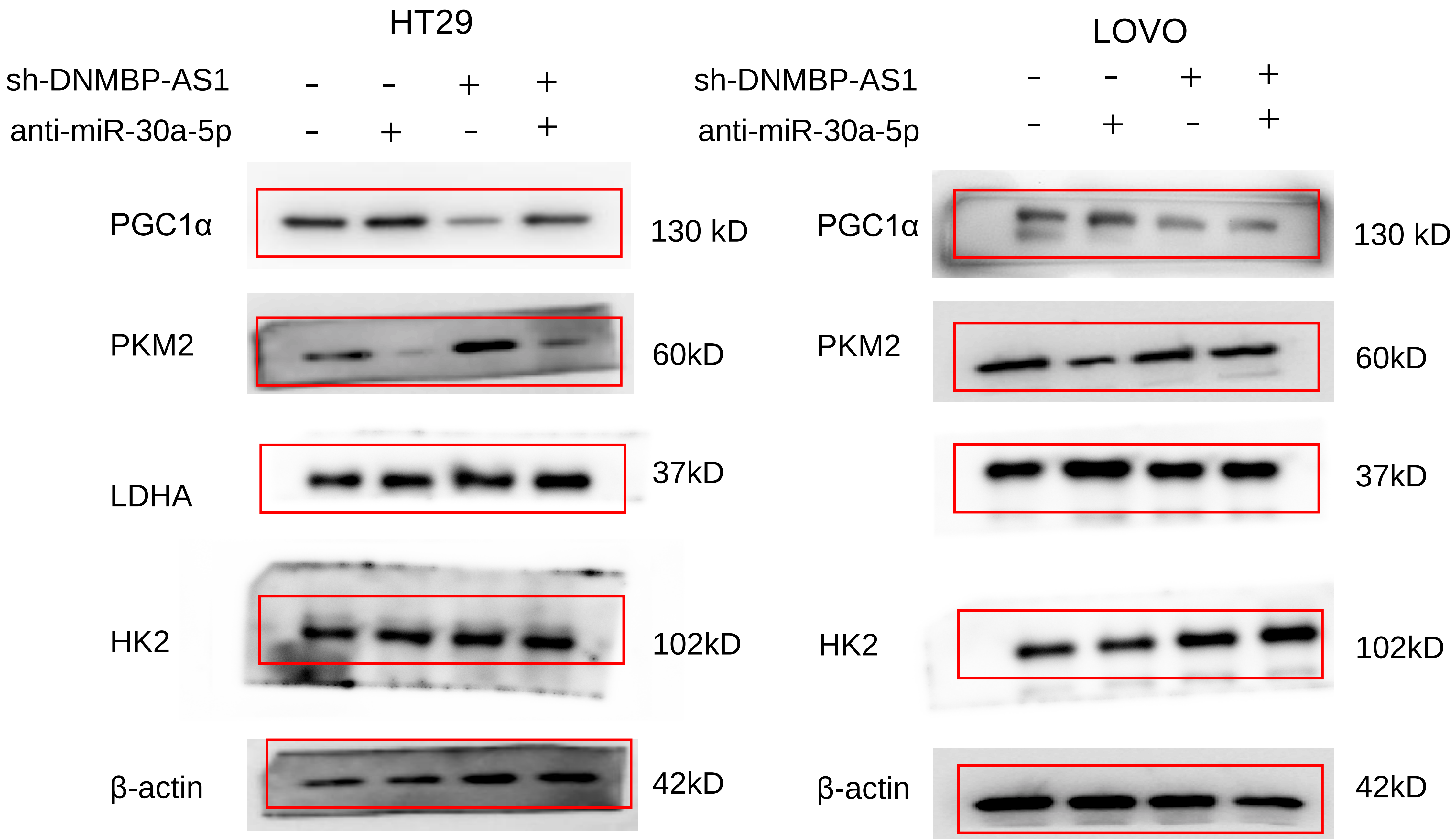

Full unedited blot for Figure 7D-E

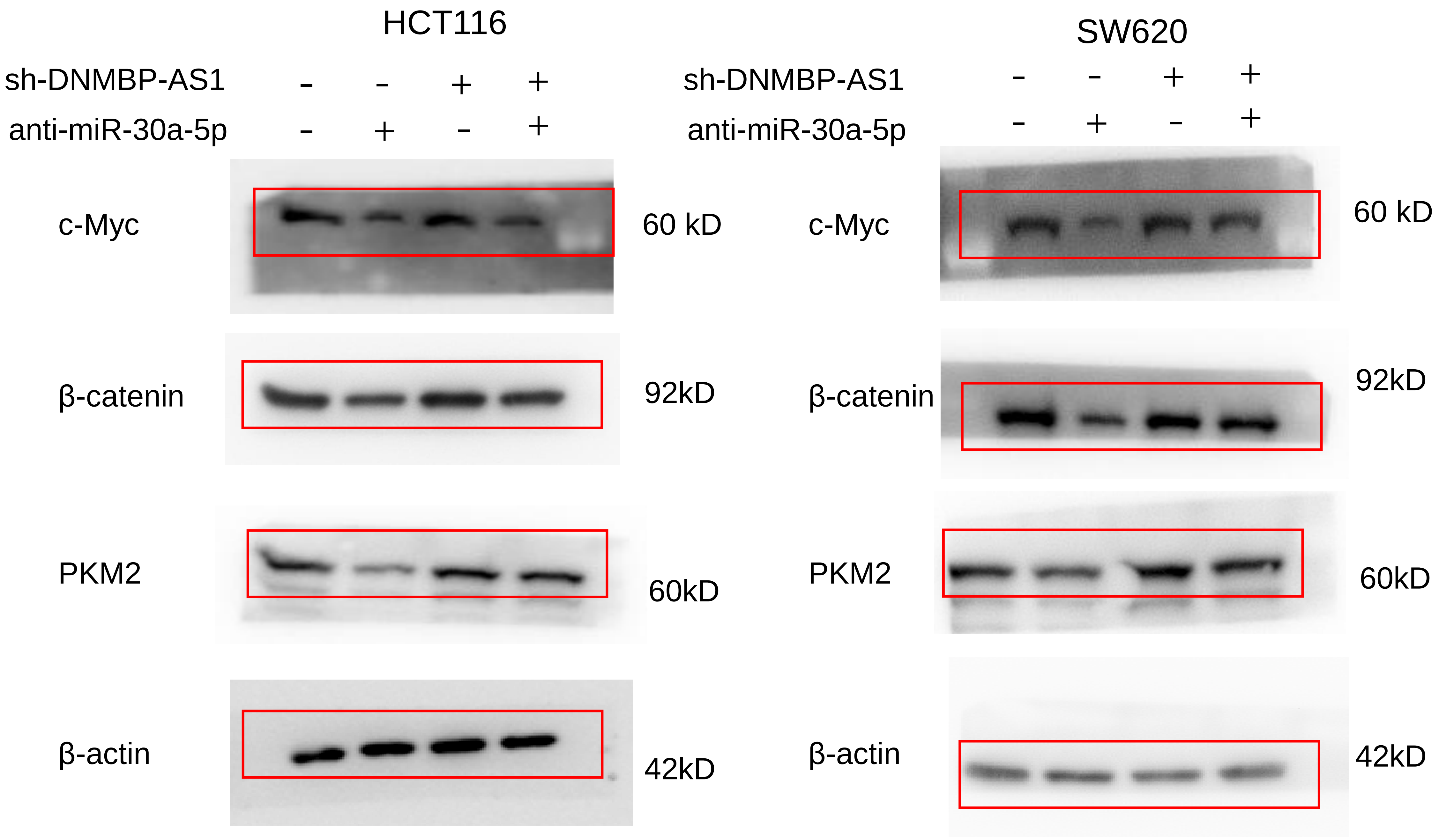

Full unedited blot for Figure 7E

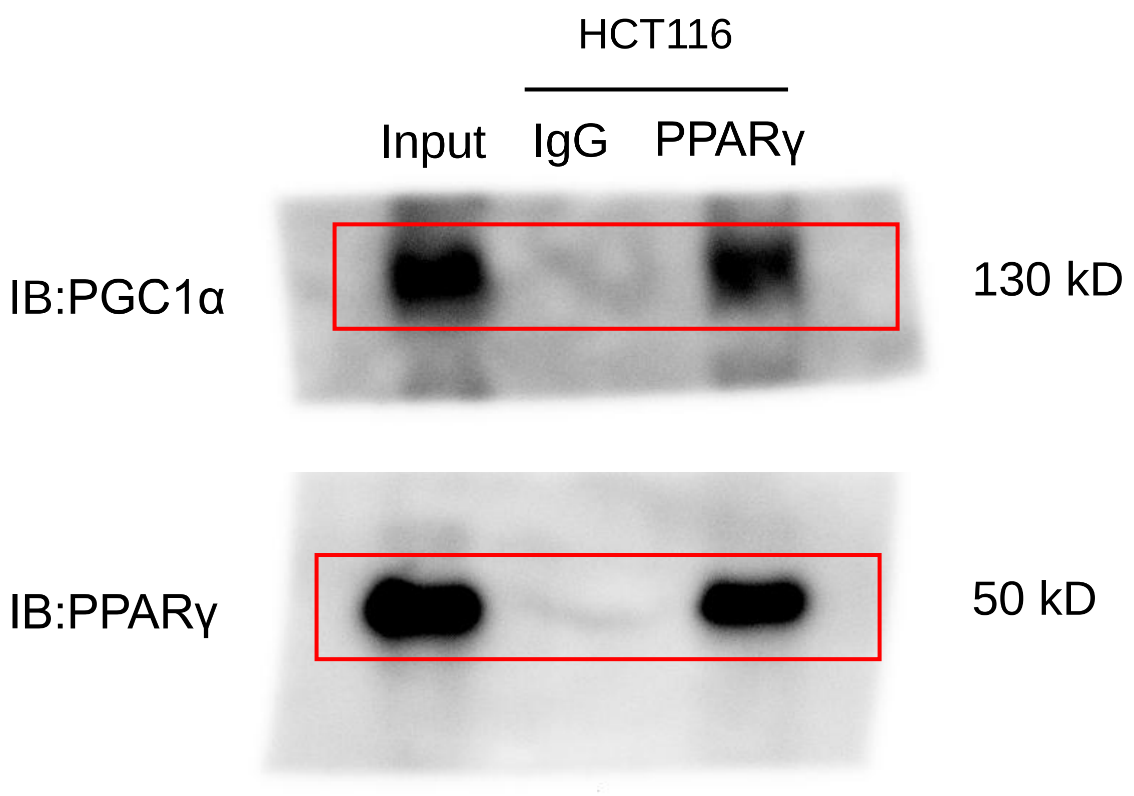

Full unedited blot for Figure 7N

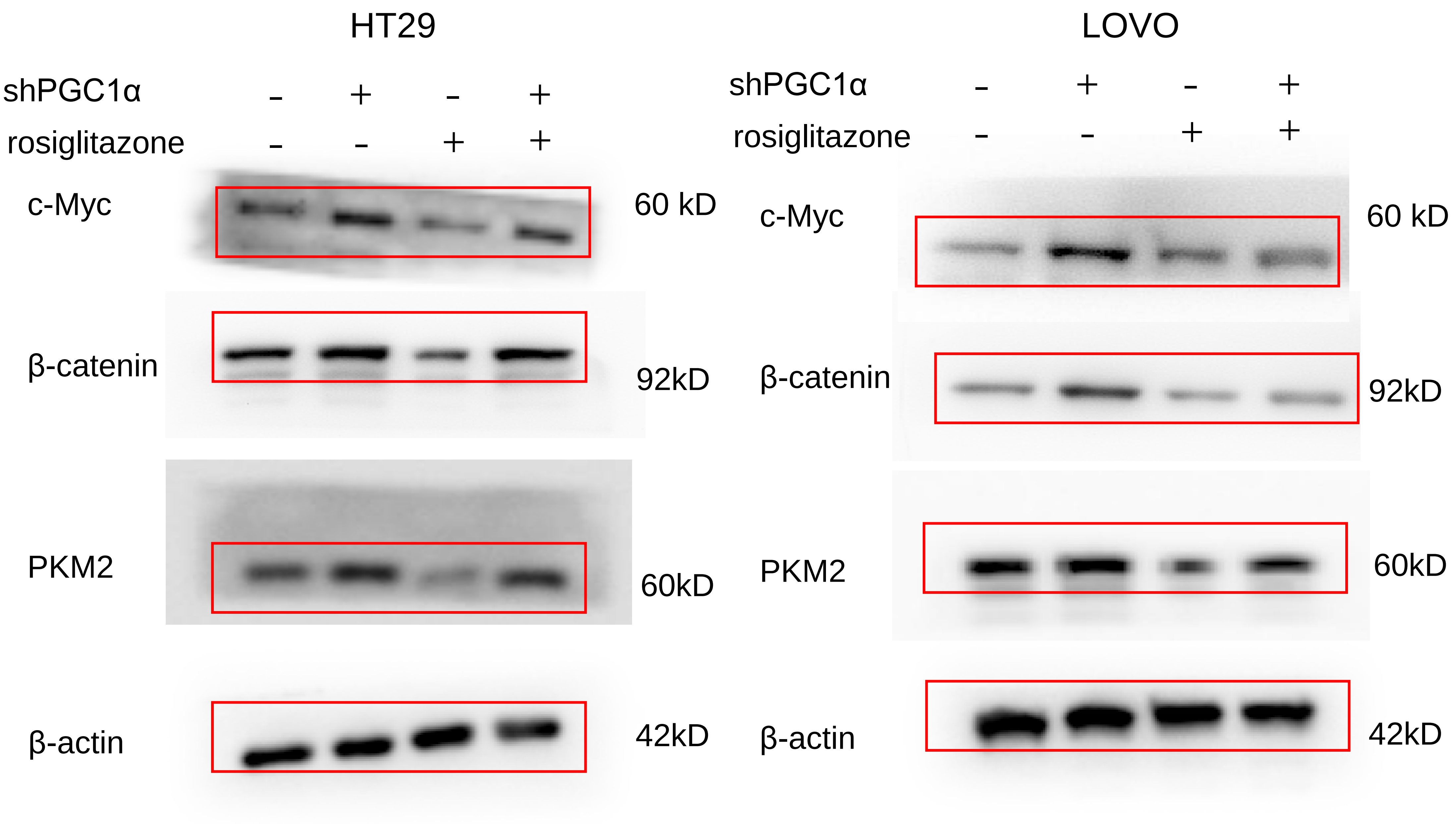

Supplement: Supplementary file 1 — supplementary materials [file 41420_2025_2561_MOESM1_ESM.pdf]
