# Supplementary material for: DNMBP-AS1/hsa-miR-30a-5p/PGC1α axis suppresses tumor progression of colorectal cancer by inhibiting PKM2-mediated Warburg effect and enhance anti-PD-1 therapy efficacy
Source: Cell Death Discov. 2025 Jul 2;11:299. doi: 10.1038/s41420-025-02561-2 (PMC12222716; doi:10.1038/s41420-025-02561-2)
Supplement: Supplementary file 2 — Supplementary Code File 1 [file 41420_2025_2561_MOESM2_ESM.pdf]

```
library(mlr3verse)

library(mlr3)

library(dplyr)

library(tidyr)

library(DataExplorer)

library(ggplot2)

library(gridExtra)

library(precrec)

library(mlr3viz)

library(GGally)

library(missForest)

rm(list=ls())


setwd("D:/ ")

COAD <- read.csv(file = "TCGA_clinical rev_5year_rev.csv",header = T,na.strings ="")

COAD <- COAD[,-1]

for (i in colnames(COAD)[1:9]){

  COAD[,i]<-as.factor(COAD[,i])

}

COAD_add <- missForest(COAD,ntree = 500)

COAD<- as.data.frame(COAD_add$ximp)

plot_bar(COAD,ggtheme = theme_bw(), ncol = 4, nrow = 4)

plot_boxplot(COAD,by = 'event',ncol = 4, nrow = 4)


# Correlation

plot_correlation(COAD)
```

```
# Random Forest -----------------------------------------------------------
---------
set.seed(1234)
task <- as_task_classif(COAD, target = "event", positive = "1")
learner_ranger <- lrn("classif.ranger", predict_type = "prob") # Specify prediction type
learner_ranger$param_set##Determine parameter types
# Set hyperparameter types: max depth, number of trees, minimum node size
search_space <- ps(
  max.depth = p_int(lower = 1, upper = 5),
  num.trees = p_fct(seq(from=500,to=1000,by=100)),
  min.node.size=p_int(lower = 1, upper = 10)
)
search_space
terminator <- trm("evals", n_evals = 300) # Set stopping criteria
tuner <- tnr("random_search") # Select search method
resampling <- rsmp("cv") # Select resampling method
measures <- msr("classif.mcc") # Select evaluation metric
at <- AutoTuner$new(
  learner = learner_ranger,
  resampling = resampling,
  search_space = search_space,
  measure = measures,
  tuner = tuner,
  terminator = terminator
)
at$train(task)
```

```
# Create new learner with optimized parameters

learner_ranger_new <- lrn("classif.ranger", predict_type = "prob",

                              max.depth =5,num.trees=600,min.node.size=5,importance = "impurity")

resampling <- rsmp("cv", folds = 10)


RF <- resample(task, learner_ranger_new, resampling, store_models = T)

RF$score(msrs(c("classif.acc","classif.auc","classif.mcc")))[1:10]

RF <- learner_ranger_new$train(task)


prediction <- learner_ranger_new$train(task)$predict(task)

autoplot(prediction, type = "roc")

prediction$score(msrs(c("classif.acc","classif.auc","classif.mcc","classif.sensitivity","classif.specificity")))

train <- learner_ranger_new$train(task)

train$importance()

save(RF,file = "RF_COAD.Rdata")

importance = as.data.table(train$importance(), keep.rownames = TRUE)

# Modify column names

colnames(importance) = c("Feature", "Importance")

# Plot importance with ggplot

ggplot(data=importance,

          aes(x = reorder(Feature, Importance), y = Importance)) +

   geom_col() + coord_flip() + xlab("")


# XGBoost -----------------------------------------------------------------

for (i in colnames(COAD)[1:9]){
```

```
  COAD[,i]<-as.numeric(COAD[,i])
}
task <- as_task_classif(COAD, target = "event", positive = "1")
learner_xgboost <- lrn("classif.xgboost", predict_type = "prob") # Specify prediction type
learner_xgboost$param_set##Determine parameter types
# Set hyperparameter types: max depth, number of trees, minimum node size
search_space = ps(
  eta = p_dbl(lower=0,upper=1),
  gamma = p_dbl(lower=1,upper=5),
  max_depth = p_int(lower = 1, upper = 10),
  min_child_weight = p_dbl(lower = 1, upper = 10),
  subsample = p_dbl(lower = 0.5, upper = 1),
  colsample_bytree = p_dbl(lower = 0.5, upper = 1),
  nrounds = p_int(lower = 1, upper = 50)
)
search_space
design = expand.grid(eta = c(0.01, 0.1, 0.5),
                     gamma = c(1, 3),
                     max_depth=c(4, 8),
                     min_child_weight = c(3, 6),
                     subsample = c(0.6, 0.9),
                     colsample_bytree = c(0.6, 0.9),
                     nrounds = c(10, 20),
                     stringsAsFactors = F) %>% as.data.table()

tuner <- tnr("design_points", design= design) # Select search method
```

```
resampling <- rsmp("cv") # Select resampling method
measures <- msr("classif.mcc") # Select evaluation metric
at <- tune(
  learner = learner_xgboost,
  task=task,
  resampling = resampling,
  search_space = search_space,
  measure = measures,
  tuner = tuner,
  terminator = terminator
)
at$train(task)
# Create new learner with optimized parameters
learner_xgboost_new <- lrn("classif.xgboost", predict_type = "prob")
learner_xgboost_new$param_set$values=at$result_learner_param_vals
resampling <- rsmp("cv", folds = 10)
XGB <- resample(task, learner_xgboost_new, resampling, store_models = T)
XGB$score(msrs(c("classif.acc","classif.auc","classif.mcc")))[1:10]
prediction <- learner_xgboost_new$train(task)$predict(task)
autoplot(prediction, type = "roc")
prediction$score(msrs(c("classif.acc","classif.auc","classif.mcc","classif.sensitivity","classif.specificity")))
train <- learner_xgboost_new$train(task)
train$importance()
importance = as.data.table(train$importance(), keep.rownames = TRUE)
# Modify column names
colnames(importance) = c("Feature", "Importance")
```

```
# Plot importance with ggplot
ggplot(data=importance,
           aes(x = reorder(Feature, Importance), y = Importance)) +
  geom_col() + coord_flip() + xlab("")

# Support Vector Machine -----------------------------------------------------------------
for (i in colnames(COAD)[1:9]){
  COAD[,i]<-as.numeric(COAD[,i])
}
task <- as_task_classif(COAD, target = "event", positive = "1")
learner_svm <- lrn("classif.svm", predict_type = "prob") # Specify prediction type
learner_svm$param_set##Determine parameter types
# Set hyperparameter types: kernel type, degree, cost, gamma
search_space = ps(
  kernel = p_fct(c("polynomial", "radial", "sigmoid")),
  degree = p_int(lower=1,upper=3),
  cost = p_dbl(lower=0.1,upper=10),
  gamma = p_dbl(lower=0.1,upper=10),
  type = p_fct("C-classification")
)

search_space
design = expand.grid(kernel=c("polynomial", "radial", "sigmoid"),
                     degree=1:2,
                     cost=c(0.1, 1,5),
                     gamma = c(0.1, 1,5),
```

```
                                    type = "C-classification",
                                    stringsAsFactors = FALSE) %>% as.data.table()
design$degree[design$kernel!="polynomial"]=NA
design = dplyr::distinct(design)
tuner <- tnr("design_points", design= design) # Select search method
resampling <- rsmp("cv") # Select resampling method
measures <- msr("classif.mcc") # Select evaluation metric

at <- tune(
  learner = learner_svm,
  task=task,
  resampling = resampling,
  search_space = search_space,
  measure = measures,
  tuner = tuner,
  terminator = terminator
)
at$train(task)

# Create new learner with optimized parameters
learner_svm_new <- lrn("classif.svm", predict_type = "prob")
learner_svm_new$param_set$values=at$result_learner_param_vals
resampling <- rsmp("cv", folds = 10)
SVM <- resample(task, learner_svm_new, resampling, store_models = T)
SVM$score(msrs(c("classif.acc","classif.auc","classif.mcc")))[1:10]
prediction <- learner_svm_new$train(task)$predict(task)
autoplot(prediction, type = "roc")
```

```
prediction$score(msrs(c("classif.acc","classif.auc","classif.mcc","classif.sensitivity","classif.specificity")))


# K-Nearest Neighbors ----------------------------------------------------------------------
for (i in colnames(COAD)[1:9]){
  COAD[,i]<-as.numeric(COAD[,i])
}
task <- as_task_classif(COAD, target = "event", positive = "1")
learner_knn <- lrn("classif.kknn", predict_type = "prob") # Specify prediction type
learner_knn$param_set##Determine parameter types
# Set hyperparameter types: number of neighbors, distance metric, kernel type
search_space = ps(
  k = p_int(lower = 3, upper = 50), # Number of neighbors considered
  distance = p_dbl(lower = 1, upper = 10),
  kernel = p_fct(levels = c("rectangular", "gaussian", "rank", "optimal"))
)
search_space
design = expand.grid(k = c(3,5,10,20,30,40,50), # Number of neighbors considered
                     distance = c(1,3,5,7,10),
                     kernel = c("rectangular", "gaussian", "rank", "optimal"),
                     stringsAsFactors = F) %>% as.data.table()


tuner <- tnr("design_points", design= design) # Select search method
resampling <- rsmp("cv") # Select resampling method
measures <- msr("classif.mcc") # Select evaluation metric
at <- tune(
```

```
   learner = learner_knn,
   task=task,
   resampling = resampling,
   search_space = search_space,
   measure = measures,
   tuner = tuner,
   terminator = terminator
)
at$train(task)
# Create new learner with optimized parameters
learner_knn_new <- lrn("classif.kknn", predict_type = "prob")
learner_knn_new$param_set$values=at$result_learner_param_vals
resampling <- rsmp("cv", folds = 10)
KNN <- resample(task, learner_knn_new, resampling, store_models = T)
KNN$score(msrs(c("classif.acc","classif.auc","classif.mcc")))[1:10]
learner_knn_new$train(task)
learner_knn_new$predict_newdata(a)
prediction <- learner_knn_new$train(task)$predict(task)
autoplot(prediction, type = "roc")
prediction$score(msrs(c("classif.acc","classif.auc","classif.mcc","classif.sensitivity","classif.s
pecificity")))


# Naive Bayes -----------------------------------------------------------------------
for (i in colnames(COAD)[1:9]){
   COAD[,i]<-as.numeric(COAD[,i])
}
```

```
task <- as_task_classif(COAD, target = "event", positive = "1")

learner_bayes <- lrn("classif.naive_bayes", predict_type = "prob") # Specify prediction type

resampling <- rsmp("cv", folds = 10)

bayes <- resample(task, learner_bayes, resampling, store_models = T)

bayes$score(msrs(c("classif.acc","classif.auc","classif.mcc")))[1:10]

learner_bayes$train(task)

prediction <- learner_bayes$train(task)$predict(task)

autoplot(prediction, type = "roc")

prediction$score(msrs(c("classif.acc","classif.auc","classif.mcc","classif.sensitivity","classif.specificity")))


# Linear Discriminant Analysis ---------------------------------------------------------------------

task <- as_task_classif(COAD, target = "event", positive = "1")

learner_lda <- lrn("classif.lda", predict_type = "prob") # Specify prediction type

resampling <- rsmp("cv", folds = 10)

lda <- resample(task, learner_lda, resampling, store_models = T)

lda$score(msrs(c("classif.acc","classif.auc","classif.mcc")))[1:10]

learner_lda$train(task)

prediction <- learner_lda$train(task)$predict(task)

autoplot(prediction, type = "roc")

prediction$score(msrs(c("classif.acc","classif.auc","classif.mcc","classif.sensitivity","classif.specificity")))
```
