# Supplementary material for: DNMBP-AS1/hsa-miR-30a-5p/PGC1α axis suppresses tumor progression of colorectal cancer by inhibiting PKM2-mediated Warburg effect and enhance anti-PD-1 therapy efficacy
Source: Cell Death Discov. 2025 Jul 2;11:299. doi: 10.1038/s41420-025-02561-2 (PMC12222716; doi:10.1038/s41420-025-02561-2)
Supplement: Supplementary file 4 — supplementary results [file 41420_2025_2561_MOESM4_ESM.docx]

**Supplementary results**


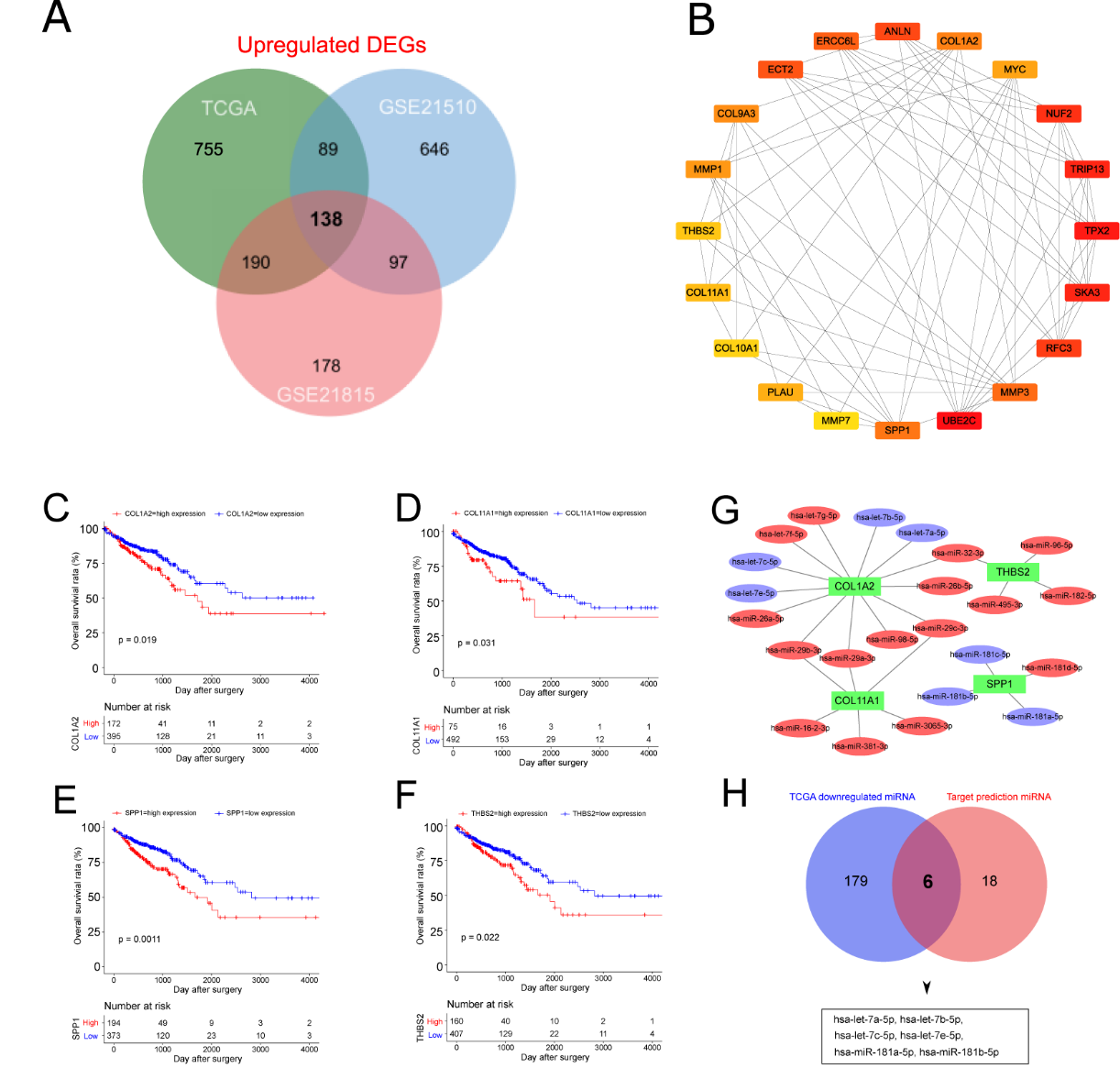


**Figure S1.** (A) The Venn diagram shows the intersection of upregulated genes in three datasets. (B) The PPI network shows the top 20 hub genes of significantly upregulated genes. (C-F) Overall survival of four upregulated mRNA in TCGA-COAD/READ cohort. (G) Construction of a miRNA-mRNA network. The blue and red ellipses represent miRNA. The green diamond indicates upregulated hub mRNA. (H) Venn diagram shows the intersection between target prediction miRNA (upstream miRNAs of upregulated hub genes) and TCGA upregulated miRNAs.


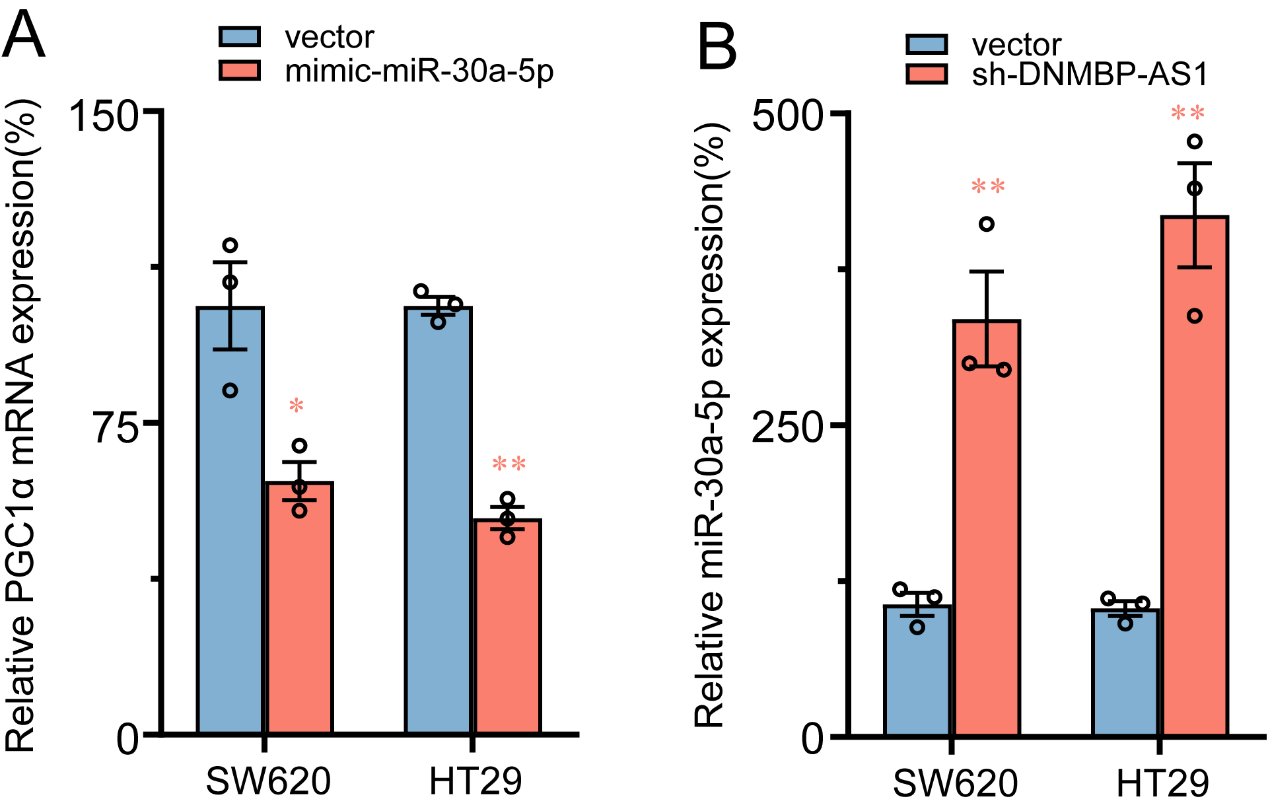


**Figure S2** Expression levels of PGC1α in SW620 and HT29 cells treatment with mimic-miR-30a-5p (A) and sh-DNMBP-AS1 (B). Statistical tests: unpaired two-tailed Student’s t-test. All data are shown as the mean ± SD of 3 independent experiments. **P< 0.05.


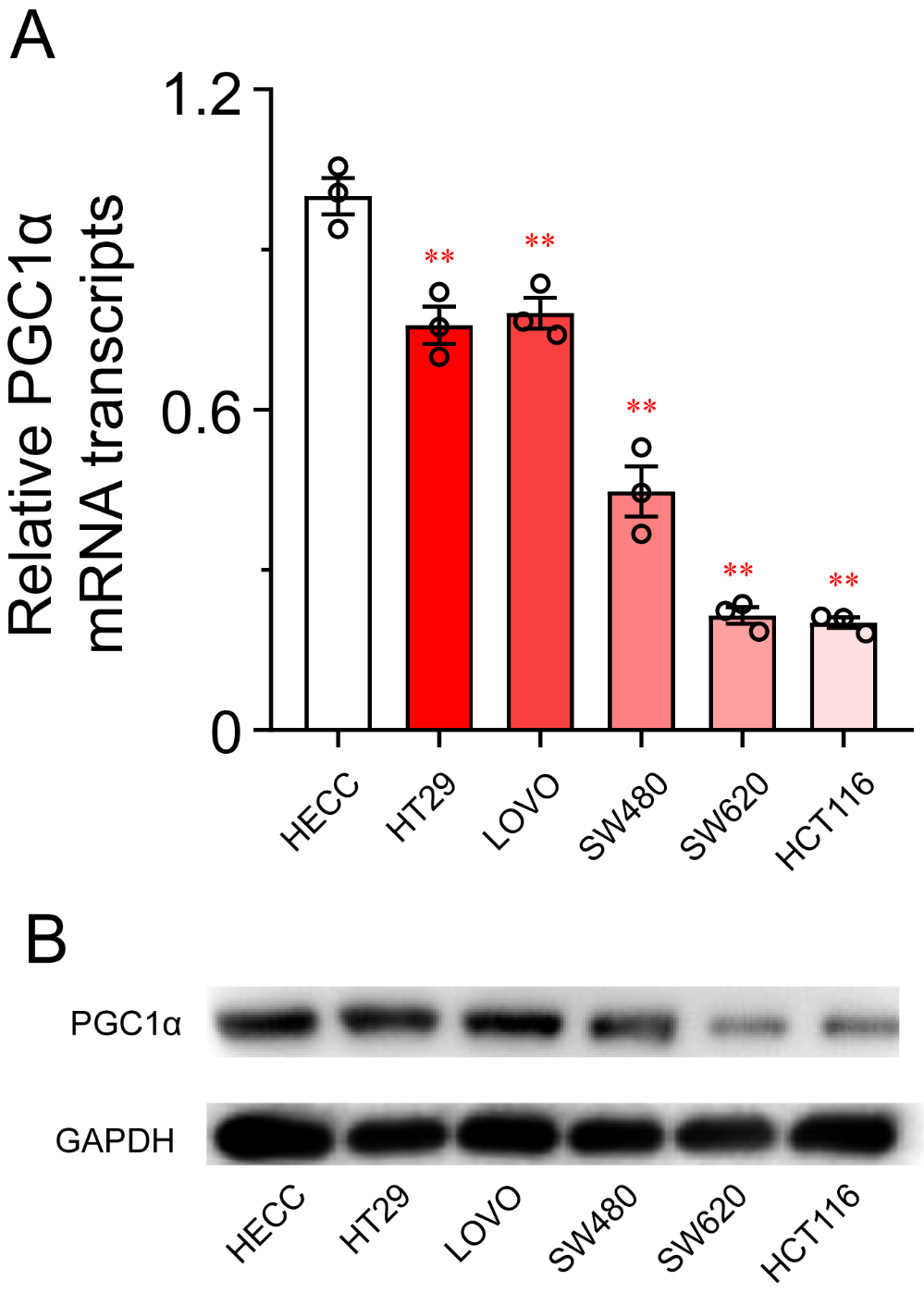


**Figure S3** Expression levels of PGC1α in CRC cell lines. The expression levels of PGC1α were measured by real-time PCR analysis (A) and western blot analysis (B) in a panel of CRC cell lines. All data are shown as the mean ± SD of 3 independent experiments. *P< 0.05, **P< 0.01


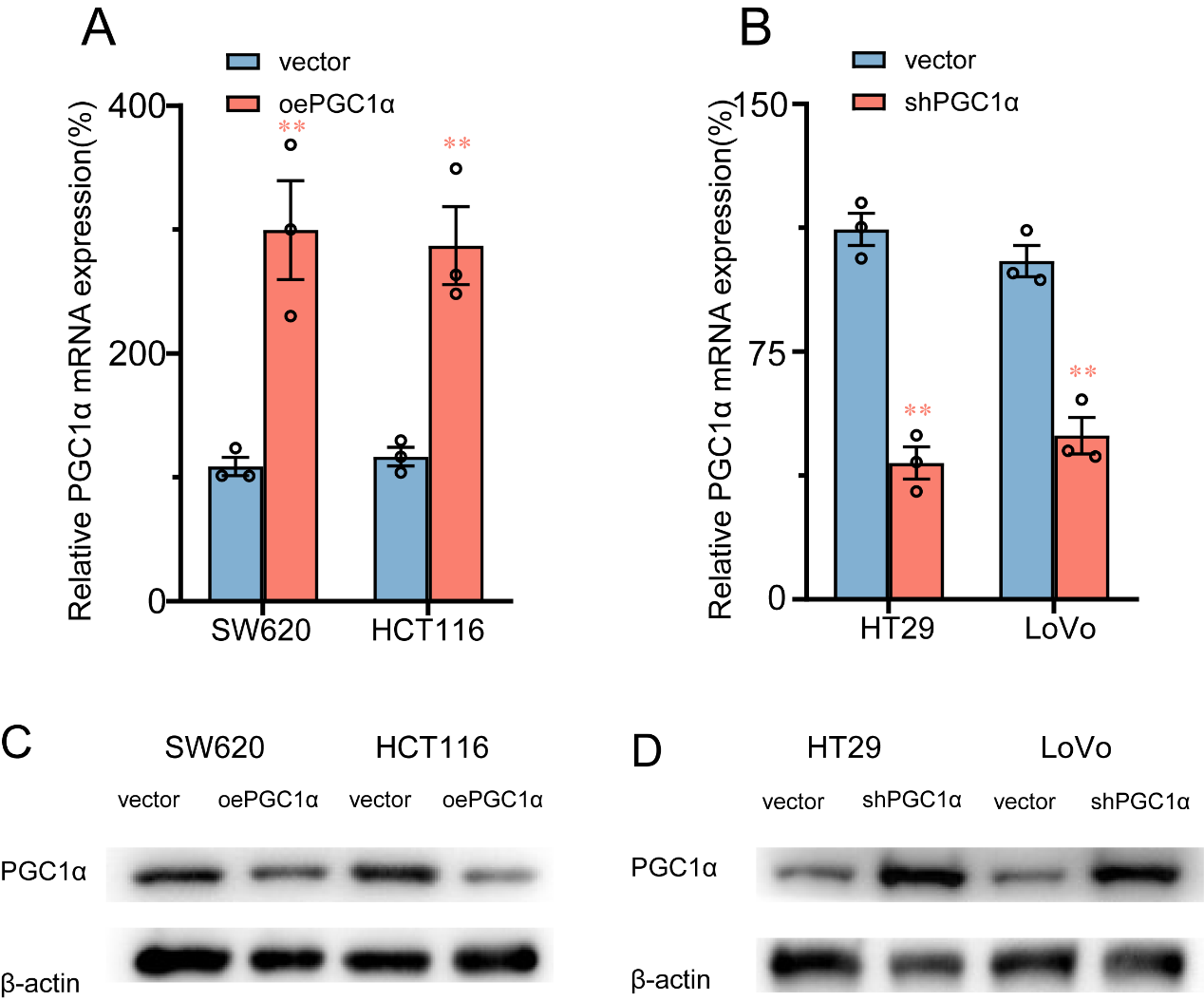


**Figure S4**. Expression levels of PGC1α in CRC cells stably infected with recombinant lentivirus. mRNA levels (A, B) of PGC1α were detected by real-time PCR in CRC cells infected with PGC1α overexpression or control lentivirus (A), PGC1α knockdown or control lentivirus (B). Protein levels (C, D) of PGC1α were detected by western blot to detect the overexpression or knockdown efficiency in CRC cells. Statistical tests: unpaired two-tailed Student’s t-test (A-B). All data are shown as the mean ± SD of 3 independent experiments. **P< 0.05.


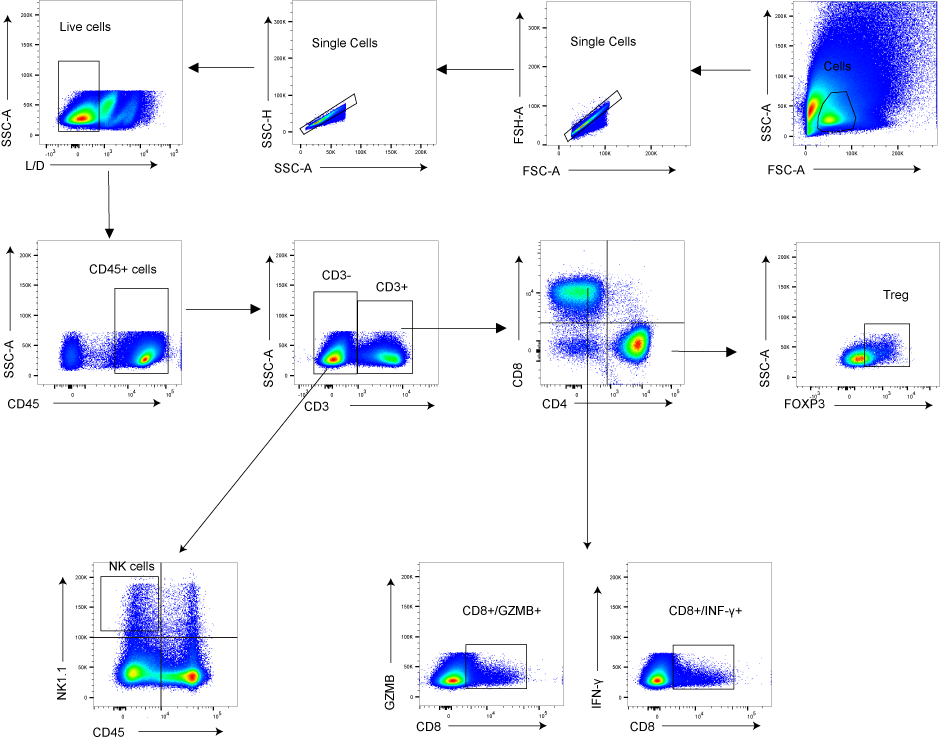


**Figure S5** Gating strategy for flow cytometry analysis of lymphoid and myeloid population in MC38 tumors

TableS1 The expression correlation between 18 miRNAs and their target mRNAs in TGCA database

| **mRNA** | **miRNA** | **cor-test** | **adj-pvalue** |
| --- | --- | --- | --- |
| PGC1α | hsa-miR-30e-5p | -0.40082 | 1.74E^-24^ |
| SLC4A4 | hsa-miR-203a-3p | -0.26518 | 4.41E^-11^ |
| PGC1α | hsa-miR-23a-3p | -0.25322 | 3.34E^-10^ |
| SLC4A4 | hsa-miR-548f-3p | -0.22636 | 1.00E^-07^ |
| SLC4A4 | hsa-miR-23a-3p | -0.22053 | 5.07E^-08^ |
| PGC1α | hsa-miR-30a-5p | -0.21657 | 8.86E^-08^ |
| SLC4A4 | hsa-miR-651-5p | -0.18683 | 1.35E^-05^ |
| PGC1α | hsa-miR-32-3p | -0.18634 | 1.39E^-05^ |
| CA2 | hsa-miR-23a-3p | -0.16511 | 0.000119 |
| PGC1α | hsa-miR-374b-3p | -0.15393 | 0.00037 |
| PGC1α | hsa-miR-30b-5p | -0.08533 | 0.051234 |
| SLC26A3 | hsa-miR-494-3p | -0.07399 | 0.092279 |
| SLC4A4 | hsa-miR-153-3p | -0.02988 | 0.513182 |
| SLC4A4 | hsa-miR-223-3p | -0.0119 | 0.77825 |
| PDK4 | hsa-miR-9-5p | 0.092898 | 0.033197 |
| COL1A2 | hsa-let-7e-5p | 0.575537 | 1.73E^-52^ |
| SPP1 | hsa-miR-181b-5p | -0.05193 | 0.244081 |
| COL1A2 | hsa-let-7a-5p | 0.411108 | 9.14E^-25^ |
| COL1A2 | hsa-let-7c-5p | 0.465943 | 1.89E^-32^ |
| SPP1 | hsa-miR-181a-5p | -0.06393 | 0.152212 |
| COL1A2 | hsa-let-7b-5p | 0.054938 | 2.17E^-01^ |

| **miRNA** | **lncRNA** | **cor-test** | **adj-pvalue** |
| --- | --- | --- | --- |
| hsa-mir-30a-5p | ZNF571-AS1 | -0.09131 | 0.035776 |
| hsa-mir-30a-5p | OIP5-AS1 | -0.26332 | 6.09E^-11^ |
| hsa-mir-30a-5p | DNMBP-AS1 | -0.23277 | 8.44E^-09^ |
| hsa-mir-30a-5p | LINC01133 | -0.20675 | 3.39E^-07^ |
| hsa-mir-30a-5p | ST20-AS1 | -0.17118 | 5.42E^-05^ |
| hsa-mir-30a-5p | ACAP2-IT1 | -0.16694 | 8.22E^-05^ |
| hsa-miR-23a-3p | ZSCAN16-AS1 | -0.46169 | 6.62E^-32^ |
| hsa-miR-23a-3p | OIP5-AS1 | -0.41596 | 2.02E^-26^ |
| hsa-miR-23a-3p | DNAJC27-AS1 | -0.33991 | 1.22E^-17^ |
| hsa-miR-23a-3p | MAGI2-AS3 | -0.3288 | 7.79E^-16^ |
| hsa-miR-23a-3p | LINC01550 | -0.30516 | 9.70E^-14^ |
| hsa-miR-23a-3p | TRG-AS1 | -0.30306 | 0.251444 |
| hsa-miR-23a-3p | LINC00472 | -0.24354 | 4.48E^-09^ |
| hsa-miR-23a-3p | LINC01579 | -0.2036 | 1.20E^-06^ |
| hsa-miR-23a-3p | LINC00473 | -0.20267 | 1.34E^-06^ |

TableS2 The expression correlation between 15 pairs miRNAs and target lncRNAs in TGCA database

| **mRNA** | **lncRNA** | **cor-test** | **adj-pvalue** |
| --- | --- | --- | --- |
| PGC1α | OIP5-AS1 | 0.579412 | 1.30E^-11^ |
| SLC4A4 | LINC01550 | 0.548219 | 3.58E^-47^ |
| PGC1α | DNMBP-AS1 | 0.493883 | 3.14E^-37^ |
| SLC4A4 | TRG-AS1 | 0.410805 | 5.20E^-25^ |
| PGC1α | DNAJC27-AS1 | 0.392437 | 8.23E^-23^ |
| PGC1α | LINC01133 | 0.383572 | 7.83E^-22^ |
| SLC4A4 | OIP5-AS1 | 0.368524 | 3.55E^-20^ |
| PGC1α | MAGI2-AS3 | 0.355571 | 8.02E^-19^ |
| PGC1α | LINC00472 | 0.318645 | 3.44E^-15^ |
| PGC1α | TRG-AS1 | 0.304882 | 5.49E^-14^ |
| SLC4A4 | LINC01133 | 0.291697 | 6.82E^-13^ |
| SLC4A4 | LINC00473 | 0.290023 | 8.63E^-13^ |
| PGC1α | LINC01550 | 0.287118 | 1.39E^-12^ |
| PGC1α | ZSCAN16-AS1 | 0.274626 | 1.30E^-11^ |
| SLC4A4 | MAGI2-AS3 | 0.272167 | 1.89E^-11^ |
| SLC4A4 | LINC00472 | 0.24775 | 1.12E^-09^ |
| PGC1α | LINC01579 | 0.223572 | 4.24E^-08^ |
| SLC4A4 | DNMBP-AS1 | 0.214513 | 1.44E^-07^ |
| SLC4A4 | ZSCAN16-AS1 | 0.211093 | 2.19E^-07^ |
| PGC1α | LINC00473 | 0.197405 | 1.26E^-06^ |
| SLC4A4 | DNAJC27-AS1 | 0.158143 | 1.08E^-04^ |
| SLC4A4 | LINC01579 | 0.043018 | 2.94E^-01^ |

TableS3 The expression correlation between 22 pairs mRNAs and target lncRNAs in TGCA database
